# The Cost of the Epistemic Step: Investigating Scalar Implicatures in Full and Partial Information Contexts

**DOI:** 10.3389/fpsyg.2021.679491

**Published:** 2021-07-19

**Authors:** Maria Spychalska, Ludmila Reimer, Petra B. Schumacher, Markus Werning

**Affiliations:** ^1^Institute for German Language and Literature I, Linguistics, University of Cologne, Cologne, Germany; ^2^Institute of Philosophy II, Ruhr University of Bochum, Bochum, Germany

**Keywords:** scalar implicature, primary and secondary implicature, epistemic step, competence assumption, alternatives, late posterior negativity, N400

## Abstract

We present the first ERP experiments that test the online processing of the scalar implicature *some* ⇝ *not all* in contexts where the speaker competence assumption is violated. Participants observe game scenarios with four open cards on the table and two closed cards outside of the table, while listening to statements made by a virtual player. In the full access context, the player makes a fully informed statement by referring only to the open cards, as *cards on the table*; in the partial access context, she makes a partially informed statement by referring to the whole set of cards, as *cards in the game*. If all of the open cards contain a given object X (Fullset condition), then *some cards on the table contain Xs* is inconsistent with the *not all* reading, whereas it is unknown whether *some cards in the game contain X* is consistent with this reading. If only a subset of the open cards contains X (Subset condition), then both utterances are known to be consistent with the *not all* implicature. Differential effects are observed depending on the quantifier reading adopted by the participant: For those participants who adopt the *not all* reading in the full access context, but not in the partial access context (weak pragmatic reading), a late posterior negativity effect is observed in the partial access context for the Fullset relative to the Subset condition. This effect is argued to reflect inference-driven context retrieval and monitoring processes related to epistemic reasoning involved in evaluating the competence assumption. By contrast, for participants who adopt the logical interpretation of *some* (*some and possibly all*), an N400 effect is observed in the partial access context, when comparing the Subset against the Fullset condition, which is argued to result from the competition between the two quantifying expressions *some cards on the table* and *some cards in the game* functioning in the experiment as scalar alternatives.

## 1. Introduction

In Gricean tradition, scalar implicatures are contents that are derived through a pragmatic mechanism that involves reasoning about the speaker's intentional and epistemic states. For instance, consider the paramount example of a scalar implicature: If a speaker says

(1) *Some of the cookies have disappeared*,

a listener is in a position to infer that

(2) *Not all of the cookies have disappeared*,

even though semantically (1) is also true if all of the cookies have disappeared. This mechanism can be described in more detail as follows: (i) the speaker chose to use the quantifier *some*; (ii) the speaker is obeying the Maxim of Quantity; (iii) if the speaker believed that a semantically stronger sentence of similar content with the alternative quantifier *all* was true, she would have said so; (iv) it follows, that she does not have a belief that the sentence with *all* is true, namely

(3) ¬*B*_*S*_(*p*),

where *p* is a sentence *all cookies have disappeared*. The latter can be the case either since the speaker believes *p* to be false or since she is not informed whether it is true. Note that (3), sometimes referred to as *primary implicature*, is not logically equivalent to sentence (2). To arrive at (2) as the speaker implied content, one needs to make an additional assumption that the speaker is informed, or at least opinionated, about the issue under discussion, i.e., she either believes the sentence with *all* to be true, or she believes it to be false (*B*_*S*_(*p*)∨¬*B*_*S*_(*p*)). Only given this so-called “competence assumption” (Sauerland, 2004; Schulz and Van Rooij, 2006; Geurts, 2010), one can infer that the speaker actually believes the alternative sentence with *all* to be false (*B*_*S*_(¬*p*)), equivalently, she takes (2) to be true (*secondary implicature*). This inference from the primary to the secondary implicature is sometimes referred to as “epistemic step” (Sauerland, 2004; Breheny et al., 2013).

Practically all current theories of scalar implicatures assume that they result from some sort of interplay between relevant semantic alternatives in the lexicon, e.g., *some* and *all* in the case of quantifiers. Since Horn (1972, 1989) this mechanism has been described more formally by assuming that these alternatives can be ordered according to their semantic strength to create the so-called implicational scale: 〈*all, some*〉. The stronger quantifier *all* semantically entails the weaker quantifier *some*, but the use of *some* gives rise to the implicature that a sentence of similar content with the stronger alternative is false.

However, it has been debated whether this mechanism is indeed pragmatic in nature and involves epistemic reasoning as described above, or whether it is rather embedded in grammar (Chierchia, 2004; Chierchia et al., 2011). In the grammatical account by Chierchia et al. (2011), it is argued that scalar implicatures arise as an effect of inserting the silent exhaustification operator (*exh*) of semantics similar to *only*, that acts directly on the scalar alternatives. Consequently, sentences such as *Some As are B* are ambiguous between two readings which correspond to two distinct logical forms, namely the literal, existential reading *There are As that are B* (paraphrased as *Some and possibly all As are B* and referred to as the *logical reading*) and the strengthened reading that corresponds to the secondary implicature: *There are As that are B but not all As are B* (paraphrased as *Only some As are B*). Under this view, the exhaustification occurs as obligatory once scalar alternatives are contextually active. The role of pragmatics is then reduced to activating the alternatives, for instance, based on the contextual access, and this process is optional.

The main difference between the more traditional, neogricean theories and the grammatical ones seems to concern the very nature of scalar implicatures, namely, the role of pragmatics in their derivation. From this perspective, the role of epistemic reasoning should be considered of most importance. This epistemic component in deriving scalar implicatures can be investigated by considering contexts where the speaker lacks access to full information that would allow her to evaluate the sentence with the stronger alternative (*partial access contexts*). Under the neogricean view, in such contexts, only the primary implicature can be inferred, namely, that the speaker does not hold a believe that a sentence with *all* is true (henceforth referred to as the *weak pragmatic reading*). Since this implicature is then trivially satisfied, no further strengthening is possible, as it would lead to contradiction. By contrast, under the grammatical view, the reading corresponding to the secondary implicature (henceforth referred to as the *strong pragmatic reading*) is available as an alternative logical form. As it does not depend on the Gricean mechanism described above, it is also available in partial access contexts. Importantly, in contexts with fully informed speakers (*full access contexts*), the weak and strong pragmatic readings cannot be differentiated, since if the speaker competence can be assumed, the *some but not all* reading is compatible with both accounts. This interpretation is then, in the literature, simply referred to as the *pragmatic reading*.

At face value it appears that partial access contexts provide a straightforward test between the neogricean and grammatical view; however, the issue is more subtle. Whereas, the former predicts that strong pragmatic readings are unavailable in such contexts, the latter has less specific predictions. The *exh* operator is considered mandatory “if the scalar alternatives are contextually active” (cf. Chierchia et al., 2011), but it is not clear what it means for the alternatives to be active. In particular, a proponent of the grammatical view may argue that in partial access scenarios the activation of scalar alternatives is inhibited, resulting in the endorsement of the logical interpretation. In this case, the grammatical view would not predict strong pragmatic readings in such contexts either. Furthermore, the reading with (only) the primary implicature is possible under the grammatical view as well, namely, either as an alternative logical form or as a pragmatic enrichment of the logical reading resulting from the neogricean mechanism (cf. Fox, 2014; Dieuleveut et al., 2019). In particular, the grammatical view does not deny that pragmatic mechanisms exist, rather it postulates that the *some, but not all* reading is available also as an alternative semantic parse of a sentence with *some*, and not just as a pragmatically enriched reading.

Thus, on the one hand, if one could show that strong pragmatic readings are available in partial access contexts, such a result would constitute good evidence for the grammatical theory; on the other hand if such readings cannot be found, it might not suffice to disprove this account. Therefore, it seems more promising to investigate not just the availability of the weak and strong readings on the behavioral level, but also the processing costs involved in deriving these readings. The main question is whether partial access contexts involve a processing cost that could be linked to the presumed epistemic reasoning. For instance, if the derivation of the *not all* implicature involves epistemic processes of evaluating the speaker's competence, we should see a mark of these processes both in the case when the implicature is derived, as well as in the case when it has to be canceled, respectively inhibited, due to the competence assumption violation. In the current paper we test this question by comparing the processing of pragmatically ambiguous sentences with *some* in contexts with partial and full access to the quantified domain.

### 1.1. Implicatures in Contexts of Full Information

The majority of prior research on the processing of scalar implicatures has involved experiments where full information was available to all parties involved. In these experiments, the status of the stronger alternative could be determined based on world-knowledge or a visual scenario. It has repeatedly been observed that in contexts where the strong alternative *all* is known to be true, sentences with *some*—which are then considered *underinformative*—tend to trigger divergent truth-value judgments: Their evaluation as true is taken to indicate that *some* is interpreted logically, whereas their evaluation as false indicates the pragmatic interpretation of *some*, i.e., with the scalar implicature. Such divergent truth-value judgments have been observed both for cases where the truth-value can be determined based on world-knowledge, e.g., *Some people have lungs* (Bott and Noveck, 2004), as well as in sentence-picture verification paradigms (Spychalska et al., 2016).

In addition, it has generally been assumed that if scalar implicatures are generated as results of a complex Gricean-like reasoning process, they should involve more effortful processing relative to the literal interpretation. However, both the experimental results on the implicature processing as well as the interpretation of these results have been mixed. Although, in principle both response times and eye-tracking results suggest that there exists a processing cost related to scalar implicatures (Bott and Noveck, 2004; Huang and Snedeker, 2009; Spychalska et al., 2016), other studies showed that scalar implicatures may be processed at no cost (Grodner et al., 2010; Politzer-Ahles and Fiorentino, 2013), especially if the context sufficiently supports the pragmatic interpretation (Degen and Tanenhaus, 2014, 2015; Hartshorne et al., 2015).

To directly investigate whether the scalar implicature is processed incrementally, some studies have used event-related brain potentials (ERPs), focusing especially on the N400 component. ERPs are scalp-recorded voltage changes time-locked to trigger events, such as spoken or written words; the N400 is a negative-going shift in the ERP waveform occurring between 200 and 600ms post-stimulus onset, and usually maximal around 400 ms post-onset over the centro-parietal scalp sites (Kutas and Federmeier, 2011; Swaab et al., 2012). It is modulated by broadly-understood semantic expectancy and predictability of the stimulus, e.g., it tends to be larger for words that are semantically less appropriate or less expected in the context (Kutas and Hillyard, 1980; Kutas and Van Petten, 1994; Kutas and Federmeier, 2000). The size of the N400 is also inversely correlated with the cloze probability of the triggering word, i.e., the percentage of individuals who would continue a given sentence fragment with that word (Federmeier et al., 2007). In affirmative sentences, sentence continuations that make the sentence semantically false tend to elicit larger N400 than “true” sentence continuations (Nieuwland and Kuperberg, 2008; Nieuwland and Martin, 2012; Nieuwland, 2016; Spychalska et al., 2016). Although, the functional role of the N400 has been debated (inter alia Kutas and Federmeier, 2000; Hagoort et al., 2004; DeLong et al., 2005; Nieuwland et al., 2018), many recent accounts model the N400 in terms of probabilistically understood meaning-related predictability/expectancy of the stimulus (Lau et al., 2013; Kuperberg and Jaeger, 2015; Rabovsky et al., 2018).

The majority of ERP studies on scalar implicatures have focused on measuring the modulation of the N400 elicited by content nouns downstream from the quantifier phrase when the pragmatic interpretation of the quantifier makes those nouns more or less expected (Noveck and Posada, 2003; Nieuwland et al., 2010; Hunt et al., 2013; Spychalska et al., 2016). The hypothesis behind this approach is that if scalar implicatures are incrementally processed, they should modulate the expectation for upcoming words in the linguistic input so that words consistent with the implicature would be more expected by the processor and hence elicit smaller N400 ERPs than words that are only consistent with the semantic reading but inconsistent with the implicature. Those studies have indeed showed such an effect but only under certain conditions, for instance, for those individuals who based the truth-related evaluation of the utterance on the pragmatic interpretation (Hunt et al., 2013; Spychalska et al., 2016) or for those with specific personality traits, such as low autistic spectrum quotient (AQ) (Nieuwland et al., 2010).

For instance, in the ERP experiment by Spychalska et al. (2016) (see also Hunt et al., 2013, for a similar design), participants were asked to truth-evaluate sentences such as *Some/All pictures contain Xs* with respect to visual displays consisting of five pictures and containing two categories of objects: one of them was presented in each of the pictures, whereas the other one only in two or three out of all five pictures. An example scenario displayed five pictures, each of them containing an image of a cat on the left hand side and three of them containing an additional image of a ball on the right hand side. In such a scenario, *Some pictures contain cats* is true but inconsistent with the implicature (*Some-Infelicitous* condition), whereas *Some pictures contain balls* is true and consistent with the implicature (*Some-True* condition). For those participants who predominately evaluated Some-Infelicitous sentences as false (they were labeled as *pragmatists*), a biphasic ERP effect (an N400 followed by a P600) was observed for sentence-final nouns in this condition relative to the Some-True condition. No similar effect was found for those participants who predominately evaluated Some-Infelicitous trials as true (*logicians*). Thus, the processing patterns were fully determined by the reading of *some* as reflected in the participants truth-value judgements: If the scalar implicature was taken as part of sentence truth-conditions, then its processing was incremental as indicated by the observed N400 effect.

It is also interesting that in the study by Spychalska et al. (2016), a P600 effect was observed in addition to the N400. The P600 is a long-lasting, late positive shift in the ERP wave, maximal around 600 ms post-onset over centro-parietal sites. It occurs in response to syntactic errors or complexities (Osterhout and Holcomb, 1992; Hagoort et al., 1993; Osterhout et al., 1994), but also other linguistic irregularities (Kuperberg et al., 2003; Hoeks et al., 2004; Van Herten et al., 2005; Bornkessel-Schlesewsky and Schlesewsky, 2008), as well as some pragmatic violations (Chevallier et al., 2010; Regel et al., 2011). It has been argued to reflect combinatorial aspects of linguistic processing (Kuperberg, 2007), inferential processing (Burkhardt, 2006; Schumacher, 2014), or even semantic integration mechanisms (Brouwer et al., 2012). In the study by Spychalska et al. (2016), the observed P600 was argued to reflect truth-related reprocessing of the sentence.

### 1.2. Implicatures in Contexts of Partial Information

Thus, far, only a few studies have attempted to test the role of the speaker's epistemic state for the listener's interpretation of pragmatically weak statements. Goodman and Stuhlmüller (2013) run online questionnaires using scenarios where the speaker, who had either a complete or partial access to the model, made a statement using the quantifier *some*, e.g., *I have looked at 2 out of 3 letters (partial access)/3 out of 3 letters (complete access). Some of them have checks inside*. Participants were asked to estimate, by means of betting, the number of letters that had checks inside. A diminution of the pragmatic interpretation of *some* was observed in contexts where the speaker had only a partial access to the scenario: Whereas in the complete access condition, bets on 3 letters were much lower than bets on 2 letters, which shows that the implicature was derived, in the partial access condition no difference between bets on 2 and 3 letters was observed. In a self-paced reading study, Bergen and Grodner (2012) also showed a suppression of the pragmatic interpretation of *some* in situations where the speaker was assumed to have only partial knowledge. These results are generally in line with the neogricean view on scalar implicatures. However, Dieuleveut et al. (2019) provide some evidence in support of the grammatical account: Using a sentence-picture verification questionnaire, they found that *some but not all* readings may also be available in contexts where the competence assumption is violated. In this study, participants were presented with two players, who had a different view of the same set of playing cards: one of the players could see all of the cards (knowledgeable player), the other one could only see a subset of the cards (ignorant player), whereas the participants could always see all of the cards. The participants were then asked to evaluate whether a given player “can say" a given sentence. For instance, in a situation where all of the cards were hearts, the neogricean view predicts that the ignorant player, who had only a partial access to the set of cards, could say that *some of the cards are hearts*, whereas the grammatical view allows for a response that “she cannot say that," which would reflect the strong pragmatic reading. Notably, Dieuleveut et al. (2019) report up to 45% of “cannot say that" responses in such cases, supporting the grammatical account. The authors note that this result is not compatible with the traditional Gricean view, although they acknowledge that it may be reconcilable with more recent pragmatic approaches, such as the game-theoretic Rational Speech Act model (Goodman and Stuhlmüller, 2013; Bergen et al., 2016). Given that their design leaves it underspecified what kind of information the two players have about each other, specifically, what are the listener's (the other player's) beliefs about the speaker's beliefs, it could happen that the subjects engage in complex higher-order reasoning that takes into account speaker's beliefs about the listener's beliefs about the speaker's beliefs. In this case, subjects would give “cannot say that" responses not to mislead the listener about the speaker's epistemic state, more specifically, not to suggest to the other player that the speaker has full access to her cards and, thus, intends to actually communicate the scalar implicature.

Breheny et al. (2013) investigated the role of epistemic reasoning in the processing of so-called “ad hoc” (based on context-derived scales) quantity implicatures. They used a paradigm similar to the director's task known from studies on the theory of mind reasoning in *reference resolution* (Keysar et al., 2000, 2003; Nadig and Sedivy, 2002; Hanna et al., 2003; Sedivy, 2003; for a review see Noveck and Reboul, 2008). Participants were listening to a confederate speaker, who described events presented on the computer screen. The situations were observed both by the confederate speaker and the listener (i.e., subject), and consisted of an agent moving objects into two boxes, e.g., a spoon into box A, a spoon into box B, and then a fork into box A. Whereas, in the *knowledge condition* the whole sequence of actions was visible to both parties, in the *ignorance condition* the subject was aware that the speaker could not see the last action. A knowledgeable speaker could then describe the action, for instance, by saying that the agent *put a spoon into B and a spoon and a fork into A*, whereas an ignorant speaker could only say that the agent *put a spoon into B and a spoon into A*. In the latter case, the subject, who knew that nothing else was put into B (unlike into A), should have already anticipated the box B upon hearing “into" in the first subclause. Yet, subjects were aware that the speaker, who could not see the last action, could have also referred to box A in the first subclause. Using eye tracking it was shown that this information about the speaker's epistemic state modulated the listeners' processing of the speaker's utterance, namely, the listeners were delayed in anticipating the right box in the ignorance condition.

The paradigm where the listener and the speaker have a different access to the scenario has the advantage of allowing to test whether the listener takes the speaker's or egocentric perspective while interpreting the utterance. However, to distinguish between the primary and secondary implicature of *some*, it is sufficient to use a less complex paradigm, where partial access scenarios are introduced, without allowing the listener a privileged access.

Let us consider a game scenario with six cards, four of which are placed face-up, whereas two are placed face-down. All of the face-up cards are hearts but it is not known what suit the face-down cards are. Suppose now that a speaker looks at the cards and says *Some of the cards are hearts*. Under the neogricean view, it is a pragmatically felicitous statement, since it is not known whether all of the cards are hearts, thus, the primary implicature is satisfied. Under the grammatical view, the statement is ambiguous between the two readings: the logical reading and the strong pragmatic reading, i.e., the reading with the *exh* operator. If *Some of the cards are hearts* is interpreted under its strong pragmatic reading, it is not expected to be uttered in this scenario since its truth-value cannot be determined.

Note that it makes little sense to ask whether *Some of the cards are hearts* is true in this case, since such a question would bias toward the neogricean view on SIs. In this framework the statement is simply true: The logical reading of *some* is true and so is the primary implicature. In contrast, under the grammatical view, the truth-value of this statement is only determined if it is interpreted logically. Otherwise, it is unknown.

Instead of asking whether the sentence is true or false, we should rather ask whether the sentence “can be uttered,” or whether “it is appropriate for the speaker to utter it," given the information she has. It is another Gricean maxim, Maxim of Quality, that requires from the speakers to make their contribution one that is true, by not making false statements or statements for which they lack sufficient evidence. Taking the reformulation of the maxim by Gazdar (1979), the speaker is then expected to only utter statements which she knows or believes to be true. Therefore, if the listener adopts the strong pragmatic reading, she should consider *Some cards are hearts* as an “inappropriate” utterance if not all cards can be seen.

Partial access scenarios allow us to test the availability of the strong pragmatic reading; however, they do not allow us to differentiate between the primary implicature (weak pragmatic reading) and the logical reading: In such scenarios, *Some cards are hearts* is true and appropriate under both these readings. To contrast the primary implicature and the logical interpretation, full access scenarios are necessary. Suppose now that the four face-up cards are dealt to the table and the two additional face-down cards are dealt outside of the table, as the speaker's cards; however, in such a way that the speaker cannot look at them. The speaker then describes the game situation by referring either to all of the dealt cards as “cards in the game” or to the open cards only, as “cards on the table.” Any statement about the “table-cards” can be truth-evaluated; however, for statements referring to the cards in the game one has to consider also the closed cards. Thus, if the speaker refers to the cards on the table, she has full access to the quantifier domain, whereas if she refers to the cards in the game, she has only partial access.

Introducing two alternative domain restrictions allows us to compare partial with full access contexts in situations where the visual scene remains unchanged. However, the choice of such an experimental design has further consequences regarding the interplay between the alternatives in the experimental context, since the two domain restrictions function as scalar alternatives as well. *Some* is a monotone increasing quantifier in its first argument, which means that if *Some As are B* is true and A is a subset of A', then *Some A's are B* is true as well. Given that, in this specific setup, *cards on the table* is a subset of *cards in the game, Some cards on the table are hearts* entails that *Some cards in the game are hearts*, but the inverse does not hold. Accordingly, *Some cards in the game* may give rise to the implicature that *Some cards on the table* is not applicable. This inadequacy of the Table restriction must be due to pragmatic reasons, since whenever *Some cards in the game are hearts* is *known* to be true, *Some cards on the table are hearts* must be semantically true as well (since hearts must be visible in order for the former to be known). In short, *Some cards in the game are hearts* may be seen as pragmatically more appropriate in contexts where all visible cards are hearts simply because if only a subset of visible cards are hearts, the Table restriction is more informative. In fact, if all visible cards are hearts, the Game restriction can be used without making a commitment about the scalar implicature of the bare *some*. The same is the case if only a subset of the visible cards are hearts and one uses the Table restriction. Such a global pragmatic mechanism is predicted by the hypothesis that scalar implicatures are derived not only at the level of quantifier and based on a locally-given context (a given “hand" of cards) but they also arise in relation to a global context which, in this case, is created by a competition between two alternative domain restrictions that are used in the whole experimental setting.

### 1.3. The Current Study

In the current experiment, we used a paradigm similar to the above-described game-scenario to test the processing of sentences with the quantifier *some* in contexts with a partial and full access to the quantified domain. Participants were exposed to game situations and asked to judge statements of a previous player, introduced as Lena, who had recorded her descriptions of these scenarios before the experiment.

**Figure 1:**
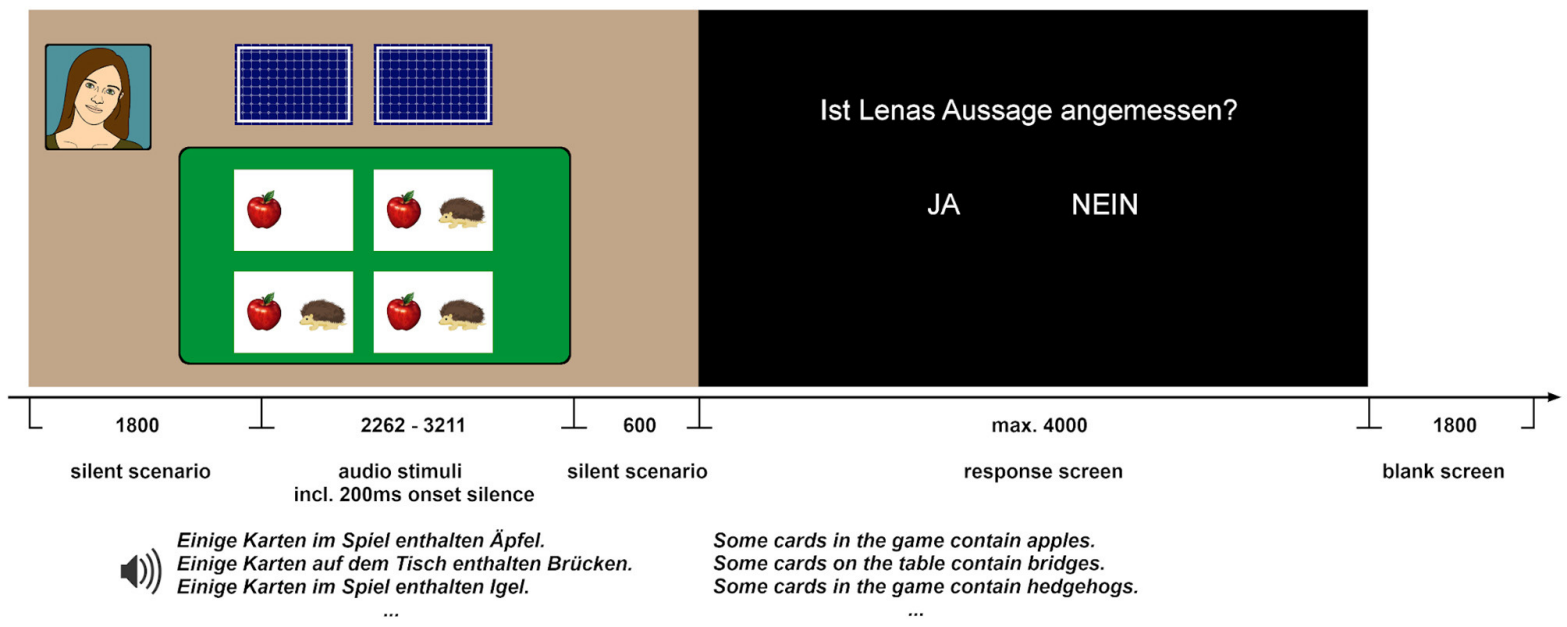
The structure of a trial: The picture was presented 1,800 ms before the onset of the audio and until 600 ms after the audio offset. The audio files varied in length (range: 2,262–3,211 ms, the average duration of Table-sentences was 2,800 ms and the average duration of Game-sentences was 2,603 ms) and contained approximately 200 ms of an initial silence for a more natural sound onset. The onset of the critical word was between 1,857 and 2,664 ms after the onset of the audio, the average onset was 2,139 ms. Subsequently, a screen with the assignment of the buttons was presented (left vs. right hand is counterbalanced). For a given scene, the condition was dependent on the specific sentence played, as explained in Table 1.

The target scenarios consisted of (i) the speaker's avatar presented at the top left part of the screen; (ii) a game table with **four open cards**; and (iii) **two closed cards** placed face-down outside of the table (Figure 1). In the target scenarios, all visible cards showed two different object categories, one in each of the four open cards (e.g., *apples* in Figure 1), and one in 2 or 3 of the open cards (*hedgehogs*). The subjects were informed that the face-down cards were also closed from the speaker's perspective, so that she had not seen what was depicted on them. The speaker's utterances were presented auditorily while the scene was displayed. The experiment employed a 2 (**Context**) × 3 (**Set**) design: The speaker's utterances either referred to the cards in the game, i.e., all cards including the face-down cards (**partial access** or **Game context:** *Some cards in the game contain As*), or to the cards on the table only (**full access** or **Table context:** *Some cards on the table contain As*). The critical noun (factor **Set**) referred to (i) the object category contained by every visible card (**Fullset** condition); (ii) the object category contained by a subset of visible cards (**Subset** condition); (iii) another object category not presented on the screen (**Unprimed** condition) (Table 1). The ERPs were measured at the onset of the critical noun.

**Table 1:** Experimental conditions for the example scene in Figure 1.

	**Set**		
**Context**	***hedgehogs* “*Igel*”**	***apples* “Äpfel”**	***bridges* “*Brücken*”**
**Table** *Some cards on the table contain*. ‘Einige Karten auf dem Tisch enthalten.'	**Subset**	**Fullset**	**Unprimed**
	true	true/false*	false
	appropriate	appropriate/inappropriate*	inappropriate
**Game** *Some cards in the game contain*. ‘Einige Karten im Spiel enthalten.'	**Subset**	**Fullset**	**Unprimed**
	true	true/unknown*	unknown
	appropriate	appropriate/inappropriate*	inappropriate

For the given scene, the condition is determined by the sentence context (Context factor: Table vs. Game) and the critical noun (Set factor: Fullset/Subset/Unprimed). For each condition, the table provides the truth-value of the target sentence as well as the expected appropriateness judgement; (*) marks the ambiguous case, where the truth-value and the response depend on the logical/weak pragmatic/strong pragmatic interpretation.

The participants were told that Lena had a task to describe the given game scenarios. Some of her descriptions were appropriate and some were not. The inappropriate statements could haven been mistakes or they could have been chosen deliberately. The subjects' task was to determine which statements of Lena were good, i.e., “appropriate” (German “angemessen”) and which were “inappropriate” (“unangemessen”)^1^. **“Inappropriate” statements** were explained to include: (i) all statements that were visibly false, such as, e.g., *No cards on the table contain frogs* in a set-up showing frogs, as well as (ii) all statements that could not be judged true or false based on the visible information, e.g., *All cards in the game contain mice* in a setting with four cards containing mice and two additional cards face-down. Only statements that were **known to be true** were **“appropriate”**. The subjects were explicitly told that Lena had seen the same set-up of cards and had no access to the content of cards that were shown backside-up^2^.

In the full access contexts, that is, if the speaker referred to *some cards on the table*, it was always possible for the subject to evaluate whether the statement was true and consistent with the implicature (Table 1): The speaker's utterances were unambiguously true in the Table-Subset condition and, hence, appropriate. They were false in the Table-Unprimed condition and as such should be judged as inappropriate. Finally, in the Table-Fullset condition, they were inconsistent with the implicature and expected to trigger divergent appropriateness judgments: A response “inappropriate” would indicate that *at least* the primary implicature was derived, whereas a response “appropriate” would indicate the logical interpretation.

By contrast, in the partial access context, that is, if the speaker referred to *cards in the game*, the truth-value could unambiguously be determined only in Game-Subset condition: In this case, the speaker's utterances were known to be true, independently of the reading of *some*, and should be judged as “appropriate” descriptions. In the Game-Unprimed condition, the truth-value was unknown, and according to the instruction, subjects should judge such descriptions as “inappropriate.” However, in the Game-Fullset condition, the speaker did not know the status of the stronger alternative *all*. Thus, the utterances were known to be true under the logical interpretation, as well as under the reading with the primary implicature, which should trigger “appropriate” judgments, but they were false and hence “inappropriate” under the strong pragmatic reading.

Consequently, three different interpretation patterns were possible in the experiment. **Logicians** would give “appropriate” judgments both in the Table-Fullset and Game-Fullset condition. Participants who derive only a primary implicature (referred to as **weak pragmatists**) would respond “inappropriate” in the Table-Fullset but “appropriate” in the Game-Fullset condition. Finally, responding “inappropriate” both in the Table-Fullset and Game-Fullset conditions would indicate that a secondary implicature was derived in spite of the speaker competence assumption being violated (**strong pragmatists**). Importantly, the strong pragmatic interpretation is inconsistent with the classical Gricean account, but predicted under the grammatical theory. Dieuleveut et al. (2019) showed that such interpretations are in principle available to the speakers.

#### 1.3.1. Predictions Regarding the Expected ERP Effects

Given that in the Unprimed conditions, unlike in the Fullset or Subset conditions, the critical nouns referred to objects that were absent in the respective scenarios and, thus, not visually primed, we expected larger N400 ERPs in the Unprimed conditions relative to the Fullset and Subset conditions, for both context types. This prediction is uncontroversial and based on the prior literature that shows a modulation of the N400 by priming (Kutas and Federmeier, 2011).

The contrast between conditions Table-Fullset and Table-Subset allows us to test the effect of the implicature violation in the full access context. From a logical perspective, this contrast corresponds to the comparison between Some-Infelicitous and Some-True conditions in Spychalska et al. (2016). Therefore, a similar ERP pattern could be expected, namely, differential effects depending on the (appropriateness) judgments given by the participants: a null effect in the case of the logical interpretation and a biphasic N400/P600 effect in the case of the pragmatic interpretation. However, one should keep in mind that the current study differs to a large extent from the prior one, including factors that potentially may affect the time-course of the implicature processing, such as (i) different modality (auditory vs. visual presentation of the linguistic stimuli) and, consequently, a different (faster) pace of the stimulus presentation, (ii) different type of judgment (appropriateness vs. truth-value) (iii) different proportion of scalar alternatives in the design (e.g., fewer trials with *all*), and finally (iv) the presence of the two different contexts (Table vs. Game) in the current design. The role of these aspects for the observed effects is discussed in more detail in the section 4.

Contrasting conditions Game-Subset and Game-Fullset allowed us to directly test the role of epistemic access for the processing of pragmatically ambiguous sentences with *some*. In the former condition, the speaker's utterances are unambiguously true and in the latter condition, their status depends on the interpretation: they are true under logical and weak pragmatic interpretation, but have an unknown truth-value under the strong pragmatic interpretation. Therefore, differential effects were expected depending on the appropriateness judgments' pattern. In principle, as the strong pragmatic reading reflects a high level of lexicalization of the implicature, the meaning-related expectancy for the critical noun should be modulated by reading and reflected in higher N400 ERPs for the condition directly inconsistent with this reading, i.e., condition Game-Fullset.

Although both the logical and weak pragmatic reading would be associated with the same “appropriate” judgments in the Game-Fullset condition, the two reading were expected to lead to differential ERP patterns. Unlike the logical reading, the weak pragmatic interpretation assumes sensitivity to the implicature, which is derived in the full access context and is either suppressed or canceled in the partial access context. Thus, for weak pragmatists, the epistemic uncertainty associated with the Game-Fullset condition was expected to lead to more effortful processing, possibly due to the evaluation of the speaker's epistemic access. Although these processes were not expected to modulate the N400 ERPs, they were expected to trigger a different type of effect. For instance, in memory research, late (and sustained) posterior negativity has been linked to (delayed) context retrieval/context monitoring/revision processes (see Mecklinger et al., 2016, for a review). By contrast, other authors link late positivity effects to inferencing and reevaluation mechanisms (cf. Burkhardt, 2006; Schumacher, 2013).

Considering the expected ERP results, one should also take into account the contrast between the two context types in the experiment. Based on the prior literature, it is known that contextually available alternatives have an effect on the observed ERP effects (e.g., Augurzky et al., 2019). Based on the scalar relation between the alternative contexts, *Some cards in the game* may be considered underinformative in the Subset condition, given that for the Subset condition a more informative expression, i.e., *some cards on the table*, is available. By contrast, in the Fullset condition, the use of the Game restriction is pragmatically more optimal than the use of the Table restriction, as it does require any commitment about the scalar implicature of the bare *some*. This interplay between the informativity of the two expressions may have an effect on the processing patters. Upon hearing *some cards in the game* the processor may expect that the object contained by all visible cards will be mentioned rather than the one contained by a subset of visible cards. Similarly, the expectation for the object contained by the subset of cards should be larger in the Table context rather than in the Game context. This may lead to larger N400 ERPs for the Game-Subset relative to the Game-Fullset as well as relative to the Table-Subset condition. Notably, this prediction goes almost directly against the expected effect that should be observed in the case of strong pragmatists. Given that this prediction is based on the assumption that the processor does not commit to the scalar implicature of bare *some*, this effect might be most pronounced for logicians.

A direct comparison between the two context types (Table vs. Game) for a given Set condition allows to contrast the role of context. The question is whether the Game context leads to generally more effortful processing due to the epistemic uncertainty aspect.

## 2. Experiment 1

### 2.1. Materials

A list of 240 German nouns and their corresponding images was used to construct the target trials. All nouns were used in their plural form, were dissyllabic, 4−9 characters long (mean: 6) and of medium corpus frequency (8–17, mean: 12.82)^3^; compound nouns were excluded. All nouns denoted concrete objects that are easy to identify in a picture and well-known to an average German speaker. For each word two sentences were recorded:

(4) *Einige Karten im Spiel enthalten Xs*.
Some cards in the game contain Xs.

(5) *Einige Karten auf dem Tisch enthalten Xs*.
Some cards on the table contain Xs.

These sentence forms were selected to be as similar as possible to the stimuli used by Spychalska et al. (2016) with the only modification being the domain restriction *in the game/on the table*. The sentences were pseudo-randomly distributed across conditions per participant. To this aim, the nouns were first organized into 240 unique triples in such a way that each word was never combined twice with the same word. These triples were then randomly assigned to conditions and used to generate visual scenarios. Consequently, throughout the experiment, each object from the set of 240 targets was shown exactly twice, but always in a combination with a different object and never on the same side (left vs. right) of the picture. The combined words were controlled with respect to their frequency (maximal difference was 4; mean difference was 1). In addition, the semantic relationship between the combined words was also weakly controlled on the basis of the Latent Semantic Analysis (LSA): since no easily-accessible LSA-evaluation tool based on German corpora existed yet, the LSA values were estimated based on English translation of words using the server http://lsa.colorado.edu/. The LSA values were kept under 0.3 (mean value: 0.092) and checked for every word pair combination within a triple. To avoid co-activation of phonetically competing strings, words with the same phonetic onsets, such as *Käfer* and *Kämme* were never combined.

There were 240 target trials (40 per condition): 80 unambiguously true (appropriate), 80 inappropriate (40 visibly false and 40 of unknown truth-value), 40 where the weak pragmatic reading would lead to the “inappropriate” answer, and 40 where the strong pragmatic reading would lead to the “inappropriate” answer.

**Figure 2:**
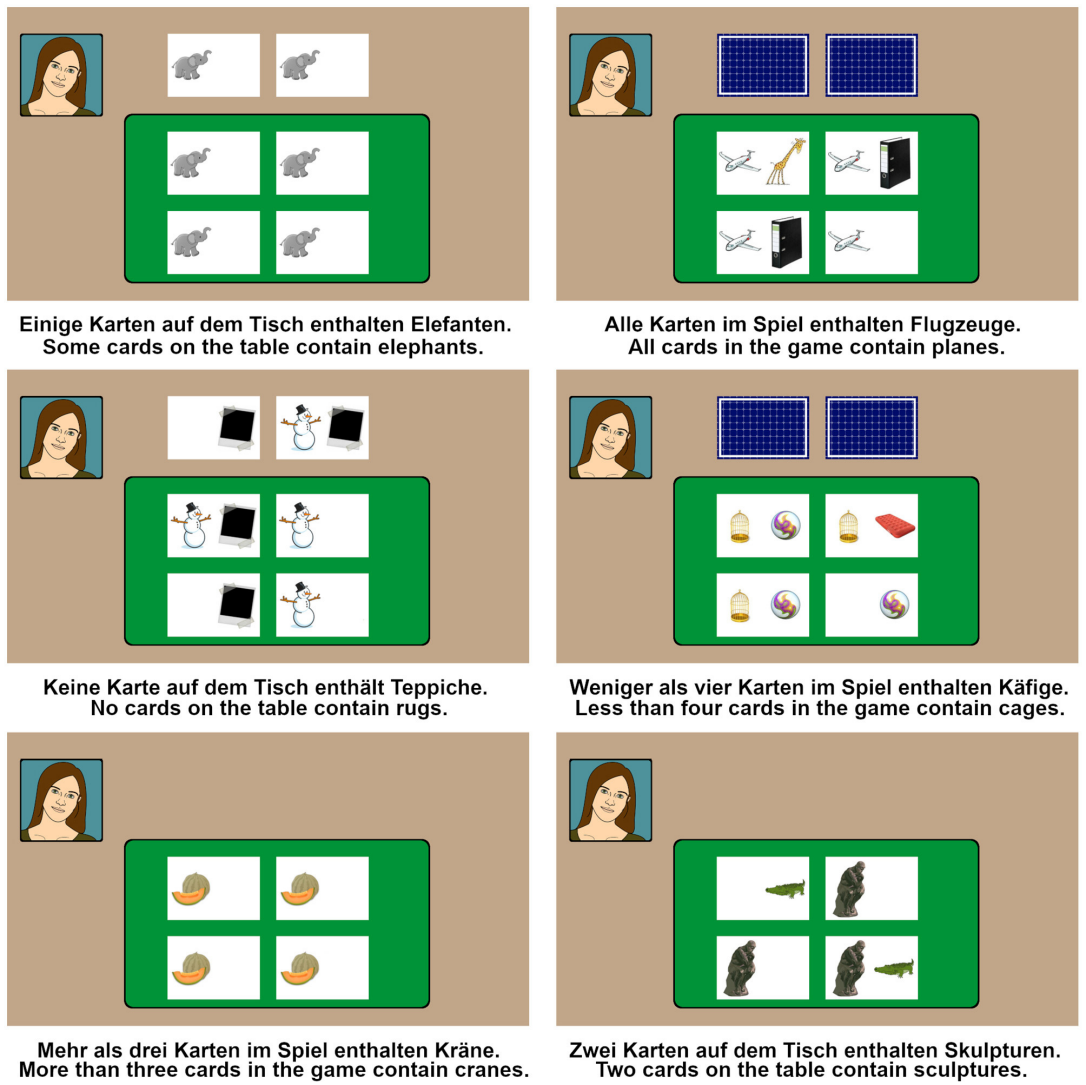
Six example filler trials of various sorts. For each quantifier all three card configurations (closed/open/no additional cards) were used. The number of visible object categories 1–3 was counterbalanced per quantifier/context/card configuration. The two contexts (Table/Game) were cross-balanced for each filler category. Sentences with bare numerals are subject to similar pragmatic ambiguity as those with *some*, i.e., based on the so-called *exactly* vs. *at least* readings. Since all target trials with *some* always showed two additional cards face-down, we introduced more fillers with two closed cards than those with other card configurations, so that the ratio of trials with closed/open/no additional cards would be relatively balanced across quantifiers.

A set of 180 filler trials was created, using additional 90 words (each filler object was shown 3–4 times, whereas each filler word was used twice auditorily). Filler trials constituted an important aspect of the design. They were used to introduce variation, reduce predictability and balance the distribution of appropriateness judgments in the experiment. Since all target trials used scenes with two closed cards outside of the table, we added filler trials with scenes where no additional cards outside of the table were dealt and scenes where these two cards were shown open. To prevent subjects from developing a strategy for predicting potential objects on the closed cards, the number of visible object categories in the filler trials was varied between 1 and 3, and subjects were explicitly instructed that the cards *may contain* 1–3 object categories. In this way, when seeing only two object categories, the subjects could not assume that there is no other category on the closed cards. The filler trials used the quantifiers: *all* (*alle*), *no* (*keine*), *more than two/three* (*mehr als zwei/drei*), *fewer than three/four* (*weniger als drei/vier*), *two/three* (*zwei/drei*), *some* (*einige*). The role of the trials with *all* was to create a contrast between *some* and its stronger alternative. Since sentences with *all* and *no* can never be visibly true in partial access scenarios (they are either visibly false or cannot be truth-evaluated), these quantifiers were also used to increase the number of (i) pragmatically unambiguous trials of unknown truth-value, as well as (ii) visibly false trials. Trials with the comparative quantifier *more than (two/three)* were introduced so that sentences with *some* were not the only ones that could be visibly true in partial access scenarios. : For instance, *More than three cards in the game contain Xs* is visibly true if there are four cards containing Xs, independently of the closed cards. Sentences with *fewer than* were used as a contrast, e.g., *Fewer than three cards contain Xs* is known to be false if four cards show Xs, independently of the closed cards. We also introduced trials with *zwei* and *drei* in order to make the materials further comparable with those from Spychalska et al. (2016), where fillers with bare numerals were used as well. Finally, fillers with *some* were used in order to vary the cards configuration and the number of shown object categories also for this quantifier. For more details see Figure 2 and Supplementary Materials.

The Game and Table contexts were counterbalanced for all trial filler types. The filler trials were constructed in such a way that the sentence appropriateness and truth-value varied across all quantifiers. Since the target trials included more appropriate trials, we constructed more inappropriate filler trials. In the whole experiment the ratio of appropriate vs. inappropriate trials was 56/44% for a logician, 45/55% for a weak pragmatist and 35/65% for a strong pragmatist.

### 2.2. Audio Recording and Preprocessing

The audio files were recorded in a professional studio at the phonetics department of the Cologne University. The speaker was instructed to read the stimuli sentences in a neutral voice without emphasizing single words. The audio recording was preprocessed with *praat*. Intervals of around 200 ms (197–203 ms) were left at the beginning of each sentence for a more natural acoustic onset, the cut-off at the end was determined by the end of the coda of the last word. The onset of the target word for every sentence was determined for sending the triggers into the EEG data file. After cutting the stimuli segments, every audio file was normalized for volume with the *audacity* software.

### 2.3. Participants

Fifty (34 women) participants were recruited for the experiment (age range: 18–42, mean: 25.14, SD: 4.86); They had at least a secondary degree (German Abitur), were German native speakers who did not learn a second language before the school age, had normal or corrected to normal vision, were right-handed, had no history of psychological or neurological problems, and were not under any medication at the time of testing. Three subjects had to be excluded since they did not complete the experiment due to an early disruption caused by technical problems with the audio. Behavioral responses from all these 47 subjects are evaluated. From the EEG analysis, one subject was excluded due to a broken electrode channel and four additional subjects due to unexpected, inconsistent, or isolated response patterns, resulting in 43 subjects used in the statistical analysis of the ERP data.

### 2.4. Procedures

The experiment was conducted in the EEG laboratory at the Philosophy Department of the Ruhr-University of Bochum. Participants were seated inside an electrically isolated and acoustically attenuated cabin, in front of a shielded glass with a computer screen behind it. The USB-powered speakers were placed inside the cabin as well as the Cedrus (USB-powered) response pad with two designated buttons.

Upon arrival every subject signed a written, informed consent of participation. They were informed that their data will be stored and handled in a fully anonymous manner, and that they have the right to withdraw from the experiment at any time. They also filled in a statement concerning their vision, medication, neurological or psychiatric history, and the Edinburgh Handedness Inventory test. Additionally, they were screened using the Autistic Spectrum Quotient (AQ) Questionnaire, two parts of the Wechsler Adult Intelligence Scale (WAIS), the Matrix Reasoning test and the digit span working memory test, and an adjusted German variant of the Standardized Reading Span Memory Test developed by Van den Noort et al. (2008).

The experiment started with an instruction presented on the screen and 8 exercise trials. The exercises included trials with quantifiers *all, no, fewer than, more than*. Game and Table contexts, the cards configuration, the number of visible object categories as well as the sentence truth-value and appropriateness were varied (see Supplementary Materials for the full list of exercise trials). Feedback was provided for the exercises: Subjects were explicitly told that the sentence is not appropriate if its truth-value is unknown, e.g., in the case of *All cards in the game contain Xs*, where X is on all visible cards, but there are two additional closed cards. No feedback was provided after the exercise session ended^4^.

Every trial started with a presentation of the scene (see Figure 1). After 1,800 ms from the onset of the scenario, the audio file was played. Triggers were sent for the onset of the trial, the onset of the audio and the onset of the sentence-final critical noun. The scenario stayed on the screen for an additional 600 ms after the offset of the audio file to allow for a more natural offset of the visual input as well as for recording undistorted ERPs up to approximately 1,000 ms post-onset the critical noun (average duration of the critical noun was 563 ms, *SD* = 78.15 ms).

### 2.5. EEG Recording and Data Preprocessing

The EEG was recorded with a 64-channel BrainAmp actiCAP EEG system. FCz location was used as the physical reference and AFz as the ground electrode. Four electrodes were relocated and used to measure eye-movement: FT9 and FT10 were used for horizontal movements (placed on the right and left temple), PO9 and PO10 for vertical movements (placed above and below the right eye). Impedance was kept below 5*kΩ*. The EEG was recorded with a sampling rate of 500 Hz, a 10 s low cut-off filter and a hardware anti-aliasing filter. The EEG data was processed using the software Brain Vision Analyzer 2.0. An off-line band-pass filter was applied: 0.1–30 Hz (order 4). Breaks and other periods of noisy signal were manually excluded. Automatic raw data inspection rejected all trials that had an absolute amplitude difference higher than 150μ*V*/150 ms or with activity lower than 0.5μ*V* per 100 ms intervals. The maximal voltage step was 50μ*V*/ms. Both vertical and horizontal eye-movements were corrected by means of independent component analysis. Data was re-referenced to the average of mastoid electrodes (TP9 and TP10). Segments from 200 ms pre-target onset until 1, 000 ms post-onset were extracted for every trial and condition. Baseline correction used the 200 ms interval preceding the onset of the stimulus. Segments with any remaining physical artifacts, including those with the amplitude lower than −90μ*V* or higher than 90μ*V*, were excluded and condition averages were calculated for each subject. The minimal number of segments that were preserved per subject and condition was 31/40.

### 2.6. Statistical Analysis

For the accuracy analysis we report descriptive statistics as well as non-parametric tests such as Friedmann test (for multiple conditions' comparisons) and Wilcoxon signed-rank tests for pairwise comparisons^5^. Kendall's W (Coefficient of Concordance) is used to estimate the effect size for the Friedman test.

For the analysis of reaction times (RTs), we performed a mixed ANOVA with *Context* (Table vs. Game) and *Set* (Fullset, Subset, Unprimed) as within-subject factors, and *Group* (2 levels: logicians vs. weak pragmatists) as a between-subject factor. For each subject the mean RTs in a given condition were computed after excluding missed and incorrect responses. Outliers (trials with response times longer than the mean in the given condition +2 standard deviations from this mean) were excluded before averaging^6^. Greenhouse-Geisser correction was applied whenever the result of the Mauchly's test indicated the violation of the sphericity assumption. For pairwise *post hoc* comparisons the *p*-values were Bonferroni corrected. Partial eta squared is reported for the effect size. For the sake of brevity, generally only the significant effects are reported.

For the statistical analysis of the EEG data we used the Matlab Fieldtrip package. We performed a non-parametric statistical procedure called cluster-based permutation test (Maris and Oostenveld, 2007; Oostenveld et al., 2011). For each subject the ERPs were averaged across trials in the compared conditions, in epochs of 0–1,000 ms post-onset and for all channels. The data-points (time × channel) between the sets were compared by a two-tailed dependent *t*-test. The significantly different (α = 0.05) data-points were then clustered according to the time-spatial adjacency. The cluster-level T-statistics were calculated by taking the sum over the *t*-values for each cluster. The cluster-level *p*-values were evaluated with a Monte Carlo simulation: For each subject the ERP averages were randomly swapped between the two conditions. The cluster-level statistics were computed again and the maximum of the cluster-level statistics was taken as the test statistics for this permutation. This procedure was repeated 10,000 times and the *p*-values of the observed cluster-level statistics were estimated as the proportion of permutations that resulted in a higher test-statistics than the observed one. This method allows one to test the significance of the effects over a broad spatio-temporal window, without having to choose any specific time-window or region, while correcting for false alarms related to the multiple comparisons. In this way one can test whether the null hypothesis that the compared conditions are exchangeable in the whole chosen epoch and the spatial region can be rejected. This method was selected due to a low level of specificity of our predictions.

We performed planned comparisons, i.e., three comparisons within the given Context level: Unprimed vs. Subset, Unprimed vs. Fullset and Fullset vs. Subset, both for the Table and Game contexts. In addition, we compared the two contexts for the same Set level (Game-Unprimed vs. Table-Unprimed, Game-Fullset vs. Table-Fullset, Game-Subset vs. Table-Subset). Given that differential effects were expected depending on the response pattern, the analysis was planned for each observed group of responders, assuming a representative sample size. Foreshadowing the results, we performed a separate analysis for the two main groups of responders (logicians and weak pragmatists).

### 2.7. The Analysis of Appropriateness Judgments

The accuracy rates (Table 2) in the unambiguous conditions Game-Subset, Table-Subset and Table-Unprimed were at ceiling level; however, the Game-Unprimed condition created difficulties for some of the subjects. In this condition the expected response is “inappropriate,” since the sentence truth-value is unknown. However, three subjects consistently (for more than 95% of trials) judged the target utterances as appropriate in this condition. Two of these subjects explained afterwards that they responded in this way since they did not want to “tell Lena” (the speaker) that she is wrong in a situation when they themselves could not know that, whereas the third subject admitted that they knew what our intended correct response was but they decided to respond differently. These three subjects were excluded from further analysis.

**Table 2:** The mean percentage (with standard deviation) of trials per condition judged as “appropriate,” after excluding occasional missed trials.

		**Table**			**Game**		
		**Fullset**	**Subset**	**Unprimed**	**Fullset**	**Subset**	**Unprimed**
**Experiment 1**							
Logicians	Approp.	98.41 (4.04)	99.02 (1.87)	0.76 (1.32)	99.09 (1.63)	99.01 (1.76)	2.57 (5.25)
(*N* = 33)	Accuracy	99.24 (1.32)	97.42 (5.25)
Weak pragmatists	Approp.	11.65 (11.45)	98.5 (1.75)	2.00 (3.87)	97.49 (2.63)	99.5 (1.05)	4.50 (12.52)
(*N* = 10)	Accuracy	88.35 (11.45)	98.00 (3.87)	95.50 (12.52)
Strong pragmatist	Approp.	0	100	0	0	100	0
(*N* = 1)	Accuracy	100		100	100		100
Other	Approp.	50.83 (46.52)	99.17 (1.44)	3.33 (3.82)	97.50 (0.0)	100 (0.0)	95.79 (2.85)
(*N* = 3)	Accuracy	96.66 (3.33)	4.21 (2.85)
**Experiment 2**							
Weak pragmatists	Approp.	05.33 (5,89)	98.98 (1.88)	1.00 (1.27)	97.62 (2.81)	99.00 (1.58)	1.14 (1.27)
(*N* = 15)	Accuracy	94.67 (5,89)	99.00 (1.27)	98.86 (1.27)
Logicians	Approp.	97.19 (4.52)	99.38 (1.77)	0.94 (1.29)	99.06 (1.86)	99.38 (1.77)	2.19 (3.39)
*N* = (8)	Accuracy	99.06 (1.29)	97.81 (3.39)
Strong pragmatists	Approp.	5.83 (5.20)	98.29 (2.96)	0	74,17 (31.35)	98.33 (1.44)	0.83 (1.44)
(*N* = 3)	Accuracy	94.17 (5.20)		100	25.83 (31.85)		99.17 (1.44)
Other	Approp.	100	100	5.00	94.87	97.50	95.00
(*N* = 1)	Accuracy	95.00	5.00

*In addition, the mean accuracy recalculated for cases where “inappropriate” is the accurate response: For conditions Table-Unprimed and Game-Unprimed, “inappropriate” responses are considered accurate; for logicians, “appropriate” is taken as accurate in condition Table-Fullset, for weak pragmatists, “inappropriate” is taken as accurate in this condition; for strong pragmatists, “inappropriate” is accurate in both the Table-Fullset and Game-Fullset conditions. In Experiment 2, strong pragmatists include 2 consistent subjects and one who was undecided between the weak and strong pragmatic interpretation. The subjects labeled as “other” responded “inappropriate” in condition Game-Unprimed, which is considered inaccurate and reflects a different and non-intended understanding of the task. These subjects showed mixed response patterns in condition Table-Fullset: In Experiment 1, there was one weak pragmatist in this group, one logician and one undecided subject; In Experiment 2, this group includes one logician*.

For the remaining subjects, the analysis of appropriateness judgments in the critical conditions revealed that the majority of subjects (33 out of 44; 75%) accepted the target sentences as appropriate in both the Table-Fullset as well as the Game-Fullset condition, which indicates the logical interpretation of *some*. These participants (logicians) were relatively consistent in their responses: between 80 and 100% of trials judged as appropriate in condition Table-Fullset, and between 92 and 100% in condition Game-Fullset. Only 11 out of 44 subjects (27.3%) rejected the target utterances as inappropriate in condition Table-Fullset, with consistency varying between-subject from 62 to 100%. Out of these 11 subjects, 10 consistently accepted the target utterances as appropriate in condition Game-Fullset (weak pragmatists) and one consistently rejected such trials as inappropriate (strong pragmatist).

We compared accuracy across conditions for logicians and weak pragmatists separately: In the Table-Fullset condition accuracy was defined according to Group: the response “appropriate” was defined as correct for logicians and the response inappropriate for weak pragmatists. For weak pragmatists, accuracy differed across conditions [χ^2^(5, *N* = 10) = 19.98, *p* < 0.001, *W* = 0.399], but there was no significant effect for logicians. Based on pairwise comparisons of the Set conditions within each context (Wilcoxon signed ranks), pragmatists' mean accuracy was lower in condition Table-Fullset compared to both Table-Subset (*z* = −2.371, *p* = 0.018, *r* = −0.433) and Table-Unprimed (*z* = −2.524, *p* = 0.012, *r* = −0.461). Also the mean accuracy in the Game-Fullset condition was lower relative to Game-Subset (*z* = −2.232, *p* = 0.026, *r* = −0.407).

Since all the subjects were screened with respect to a number of cognitive and personality traits (Table 3), we tested whether any of these values would be predictors of the response patterns. Independent *t*-tests showed no significant differences between weak pragmatists and logicians with respect to their working memory, AQ, reasoning capabilities or age (*p*>0.05 for each test).

**Table 3:** Age and gender distribution, as well as the mean values with standard deviations [*M*(*SD*)] for all cognitive tests, in percentages, per group and per experiment.

**Group**	***N***	**Gender**	**Age**	**Digit**	**Reading**	**Matrix**	**AQ**
		**men/women**		**Span**	**Span**	**Reasoning**	**total**
**Experiment 1**							
*Logicians*	33	10/23	24.61 (4.97)	10.42 (2.21)	67.61 (9.88)	11.36 (1.99)	14.82 (4.68)
*Weak pragmatists*	10	3/7	26.50 (5.29)	10.00 (2.79)	69.20 (9.72)	11.50 (1.90)	11.80 (4.32)
*Strong pragmatist*	1	0/1	28	10	67	14	22
*Other*	3	2/1	27.00 (6.25)	13.00 (6.08)	69.33 (13.05)	13.00 (1.00)	14.33 (1.53)
**Experiment 2**							
*Logicians*	8	7/1	22.75 (2.25)	10.00 (3.02)	65.5 (10.41)	n/a	16.63 (5.37)
*Weak pragmatists*	15	5/10	23.73 (4.13)	10.07 (2.25)	69.13 (10.10)	n/a	14.20 (4.90)
*Strong pragmatist*	3	1/2	23.67 (2.08)	10.00 (1.00)	66.33 (4.04)	n/a	13.33 (6.11)
*Other*	1	0/1	23	6	60	n/a	16

### 2.8. Reaction Times Analysis

**Figure 3:**
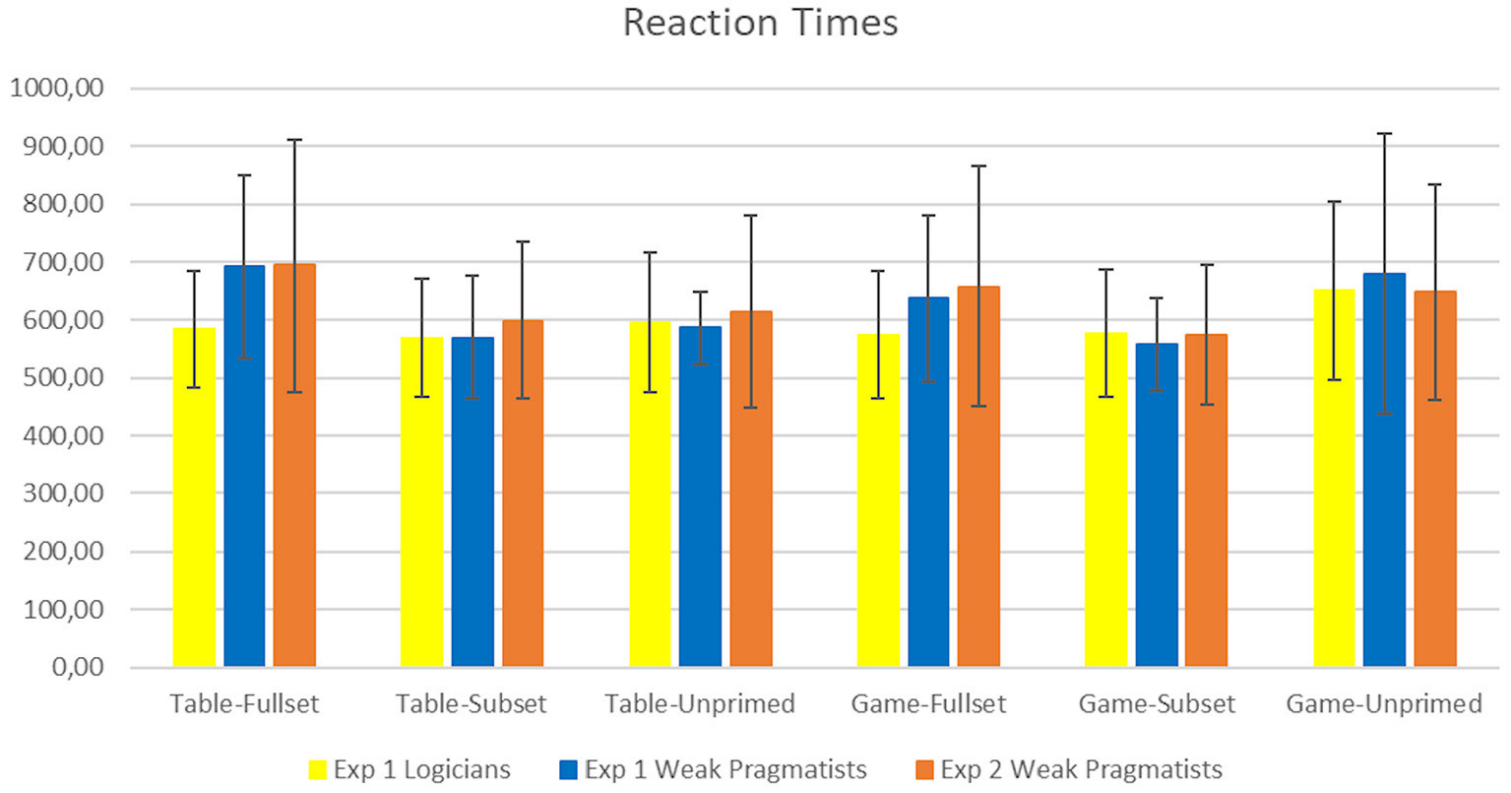
The mean response times (in ms) with standard deviations (±1 SD), for each group and condition.

The full-factorial ANOVA of the RTs (Table 4 and Figure 3) showed a significant main effect of Set [*F*(2, 82) = 15.704, *p* < 0.001, $\eta_{p}^{2}=0.277$]. The interaction Set × Context was significant [*F*(1.383, 56.700) = 19.206, *p* < 0.001, $\eta_{p}^{2}=0.319$], as well as the Set × Group interaction [*F*(2, 82) = 9.337, *p* < 0.001, $\eta_{p}^{2}=0.187$].

**Table 4:** The mean response time (with SD) in ms after excluding missed, incorrect responses and outliers (condition mean ±2 SD).

	**Table**			**Game**		
**Group**	**Fullset**	**subset**	**Unprimed**	**Fullset**	**subset**	**Unprimed**
**Experiment 1**						
*Log* (*N* = 33)	584.03 (99.70)	568.75 (102.32)	595.72 (119.95)	573.83 (109.93)	577.85 (109.36)	650.80 (153.36)
*W Prag* (*N* = 10)	691.55 (157.31)	569.74 (105.06)	586.63 (62.83)	638.07 (143.61)	558.94 (80.11)	679.29 (242.27)
**Experiment 2**						
*W Prag* (*N* = 15)	693.68 (218.35)	599.37 (134.74)	614.18 (165.44)	658.39 (208.00)	574.49 (120.56)	648.35 (186.42)

*In condition Table-Fullset, for weak pragmatists a correct response is the pragmatic response. Log = logicians, W Prag = weak pragmatists*.

The significant Context × Set interaction was subsequently broken down both by Context and by Set. First, we analyzed the RTs for each Context separately. For the Game context, there was a main effect of Set [*F*(1.356, 55.599) = 15.835, *p* < 0.001, $\eta_{p}^{2}=0.279$]. Pairwise comparisons (*p*-values were Bonferroni corrected) showed that condition Game-Subset received significantly faster responses than conditions Game-Fullset [*F*(1, 41) = 12.415, $\eta_{p}^{2}=0.232$, *p* = 0.003, Δ_*Full,Sub*_ = 37.558] or Game-Unprimed [*F*(1, 41) = 19.856, $\eta_{\text{p}}^{2}$=0.326, *p* < 0.001], Δ_*Un,Sub*_ = 96.658. The contrast between conditions Game-Unprimed and Game-Fullset was also significant [*F*(1, 41) = 11.071, $\eta_{p}^{2}=0.213$, *p* = 0.006, Δ_*Un,Full*_ = 59.100] with the former receiving slower responses.

For the Table context, there was a significant effect of Set [*F*(1.668, 68.387) = 19.722, *p* < 0.001, $\eta_{p}^{2}=0.325$] as well as a significant Set × Group interaction [*F*(1.668, 68.387) = 16.799, *p* < 0.001, $\eta_{p}^{2}=0.291$]. Pairwise comparisons (Bonferroni corrected) showed that the response times in the Table-Fullset condition were significantly longer relative to condition Table-Subset [*F*(1, 41) = 41.834, $\eta_{p}^{2}=0.505$, Δ_*Full,Sub*_ = 68.545, *p* < 0.001] and relative to condition Table-Unprimed [*F*(1, 41) = 12.268, $\eta_{p}^{2}=0.230$, Δ_*Full,Un*_ = 46.616, *p* = 0.003]. The analysis of repeated contrasts showed that the longer response times in the Table-Fullset condition were driven by the pragmatic responses: the difference between the two groups was larger in the Table-Fullset condition than in the Table-Subset condition [*F*(1, 41) = 25.266, *p* < 0.001, $\eta_{p}^{2}=0.381$, Table-Fullset Δ_*WPrag,Log*_ = 107.524 and Table-Subset Δ_*WPrag,Log*_ = 0.984], but the difference between the two groups was similar in conditions Table-Subset and Table-Unprimed (*p*>0.1).

Second, we tested the effect of Context directly for each Set condition. This step was necessary in order to test some of the missing comparisons. No effects were observed for the Subset condition. For the Unprimed conditions there was a significant effect of Context [*F*(1, 41) = 12.253, *p* = 0.001, $\eta_{p}^{2}=0.230$], here the mean response times for the Game context were longer than for the Table context (Δ_*Game,Table*_ = 73.879 ms). For the Fullset condition, the effect of Context was significant [*F*(1, 41) = 8.00, *p* = 0.007, $\eta_{p}^{2}=0.163$], namely, the Table context received on average longer response times than the Game context (Δ_*Table,Game*_ = 31.837 ms). There was also an effect of Group [*F*(1, 41) = 4.492, *p* = 0.04, $\eta_{p}^{2}=0.099$], i.e., the mean response times of weak pragmatists were longer than those of logicians (Δ_*WPrag,Log*_ = 85.884 ms).

### 2.9. EEG Results

The statistical analysis of the EEG data was performed separately for logicians (N = 33) and weak pragmatists (from the group of 10 weak pragmatists one had to be excluded from the ERP analysis due to broken electrode channels, so the number of subjects used was 9). The strong pragmatist was not included.

**Figure 4:**
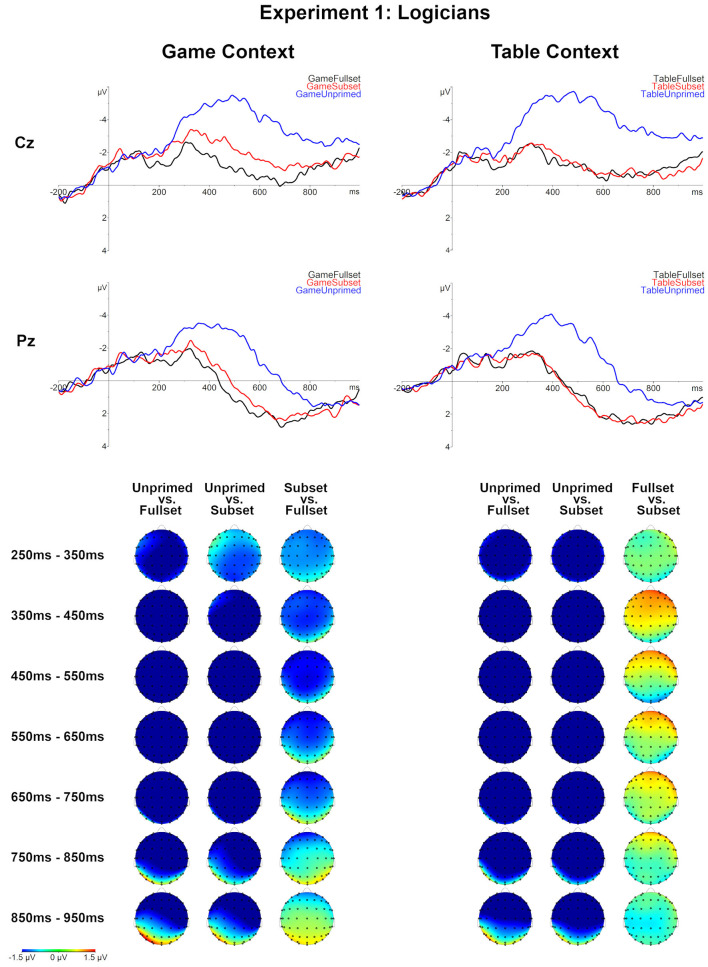
Grand averages for all conditions for logicians (*N* = 33), Experiment 1. Topographical maps of the differences between the compared Set levels separately for the Table and Game context in consecutive 100 ms time-windows, between 250 and 950 ms post-onset.

**Figure 5:**
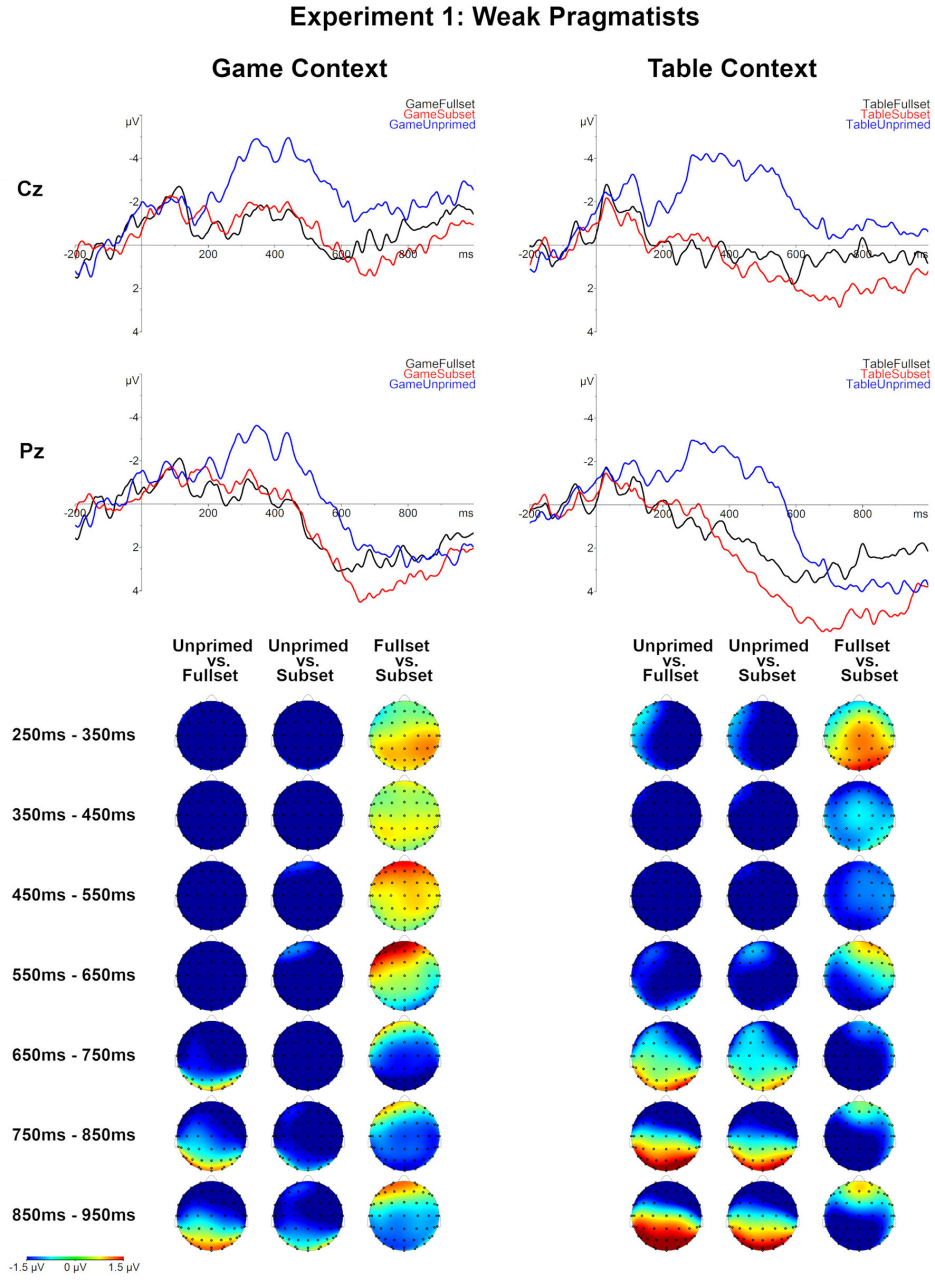
Grand averages for all conditions for weak pragmatists (*N* = 9), Experiment 1. Topographical maps of the differences between the compared Set levels separately for the Table and Game context in consecutive 100 ms time-windows, between 250 and 950 ms post-onset.

First, the three levels of the Set factor were compared for each Context level. For both contexts, a large negativity effect was observed for the Unprimed condition relative to the Subset and Fullset conditions. This effect started approximately 200 ms post-onset, lasted till the end of the epoch and had a global distribution (*p* < 0.0001 for each of the comparisons; see Table 5 and Figures 4, 5).

**Table 5:** The results of the cluster-based statistics for all comparisons: the temporal extension of the respective observed cluster (in ms) as well as its p-value evaluated by the permutation test.

		**Experiment 1**		**Experiment 2**
**Comparison**	**Cluster polarity**	**logicians (*N* = 33)**	**weak pragmatists (*N* = 9)**	***weak pragmatists* (*N* = 15)**
Table unprimed vs. Subset	Negative	214–1,000, *p* < 0.0001	224–100, *p* = 0.0032	186–1,000, *p* < 0.0001
Table unprimed vs. Fullset	Negative	150–1,000, *p* < 0.0001	186–100, *p* < 0.0001	186–1,000, *p* < 0.0001
Table fullset vs. Subset	Negative	–	[668–756, *p* = 0.068]; 784–944, *p* = 0.04	–
Game unprimed vs. Subset	Negative	266–1000, *p* < 0.0001	250–1,000, *p* < 0.0001	222–1,000, *p* < 0.0001
Game unprimed vs. Fullset	Negative	204–1,000, *p* < 0.0001	244–636, *p* = 0.0046	222–946, *p* < 0.0002
Game subset vs. Fullset	Negative	344–662, *p* = 0.0038	–	–
	positive	–	650–800, *p* = 0.025	[596–736, *p* = 0.069]
Subset game vs. Table	Negative	372–582, *p* = 0.0095	374–540, *p* = 0.0135	–
Fullset game vs. Table	Negative	–	[270–368, *p* = 0.08]; 858–100, *p* = 0.0559	612–804, *p* = 0.0074
Unprimed game vs. Table	Neg/pos	–	–	–

*Given that the weak pragmatists groups are relatively small, also marginally significant clusters are reported (0.5 < α <0.1) in []. No extra rows are added in those cases where no significant positive clusters were found*.

The comparison of the Table-Fullset and Table-Subset conditions did not show any significant effect for logicians as a separate group. However, for the weak pragmatists, this comparison lead to a marginally significant late negativity effect for the Table-Fullest condition, which based on the inspection of grand averages started around 600 ms post-stimulus onset and extended until the end of the epoch on the posterior sites: the effect's robustness is evaluated based on two adjacent clusters of 668–756 ms (marginally significant: *p* = 0.068) and 784–944 ms (*p* = 0.040).

For the Game context, differential effects are observed between the two groups. Logicians showed a negativity effect for the Game-Subset relative to Game-Fullset condition around the N400 time-window (344–662, *p* = 0.0038). By contrast, for weak pragmatists the Game-Subset vs. Game-Fullset contrast produced a positive cluster distributed over posterior channels (650–800, *p* = 0.025). The observed cluster has a positive polarity; yet, the cluster-based permutation is symmetric, so the polarity of the cluster depends on the direction of the comparison, but the polarity of the effect is a matter of interpretation. Based on the inspection of the temporal and topographical distribution of this effect, we interpret it rather as a late negativity for the Game-Fullset relative to Game-Subset condition: This effect appeared sustained until the end of the epoch and was most pronounced on the posterior sites.

Comparing the ERPs across the contexts Game vs. Table showed that the ERPs in the Game-Subset condition were more negative than in the Table-Subset condition, both for logicians (372–582, *p* = 0.0095) and for weak pragmatists (374–540, *p* = 0.0135). No significant effects were found for the comparison between conditions Table-Unprimed and Game-Unprimed. For the comparison between Game-Fullset and Table-Fullset only a marginally significant late negativity effect is observed for weak pragmatists after 800 ms post-onset (858–100 ms, *p* = 0.056) as well as a non-significant trend of an effect around the N400 time window (270–368 ms, *p* = 0.08).

## 3. Experiment 2

Given that the number of weak pragmatist in the first experiment was rather low (only 9 usable subjects), some of the observed effects could be underpowered. Therefore, we decided to run a second experiment with the aim of replicating these effects. Given that the number of logicians in the first experiment was sufficient, we decided to focus on the pragmatists only. However, we did not want to give our subjects any explicit instruction with respect to how they should interpret the sentences, as this would have significantly changed the character of the experiment. Thus, we wanted to stick to the same procedure that allowed for spontaneous responses and interpretations. Furthermore, there is no clear predictor (e.g., personality test) that would allow us to determine who will turn out to respond pragmatically in such a task. Thus, we decided to use a similar task in a form of a short questionnaire to pre-screen potential subjects with respect to their tendency to respond pragmatically or logically.

### 3.1. Pre-screening

An online questionnaire was sent out to all the participants from the lab pool who did not participate in Experiment 1 or any similar experiment. They were told that we would like them to try out a sample of tasks used in the lab experiments and that everyone will be offered a testing date, independently of their responses. The questionnaire included 16 example questions similar to those from the experiment: The same type of visual scenarios was used, but for technical reasons the sentences were presented visually under the scenarios. The examples were mainly of the filler type, but one question corresponding to the Table-Fullset condition was used. Out of 108 participants who filled out the questionnaire, 50 responded pragmatically to this critical question, which is close to the usual pragmatic vs. logical response ratio in similar experiments on scalar implicatures (e.g., Spychalska et al., 2016), and were invited to the lab for the experiment. The remaining participants were invited for other, not related to pragmatics, experiments in our lab.

### 3.2. Participants

We tested 28 participants who were pre-screened as pragmatists^7^. One participant had to be excluded due to technical problems resulting in the experiment not being completed.

### 3.3. Behavioral Results

Out of 27 subjects who finished the experiment, one gave incorrect responses in the Game-Unprimed condition. Unexpectedly, as many as 8 subjects responded logically in the Table-Fullset condition in spite of giving a pragmatic answer in the questionnaire. Out of the remaining 18 subjects who responded pragmatically in the Table-Fullset condition, 2 were consistent strong pragmatists (responded pragmatically in the Game-Fullset condition) and one had a 40/60% ratio of weak vs. strong pragmatic responses. Thus, only 15 weak pragmatists could be included in the statistical analysis, whereas the other subjects were left out (see Table 2).

Friedman test showed a marginally significant effect of condition for weak pragmatists' accuracy (in condition Table-Fullset the pragmatic response was defined as correct): χ^2^(5, *N* = 15) = 11.003, *p* = 0.051, *W* = 0.147.

### 3.4. Reaction Times

The analysis of response times (Table 4 and Figure 3) for the 15 weak pragmatists showed a main effect of Set [*F*(2, 28) = 8.758, *p* = 0.001, $\eta_{p}^{2}=0.385$]. Context × Set interaction was only marginally significant [*F*(1.42, 19.91) = 3.74, *p* = 0.055, $\eta_{p}^{2}=0.211$, based on the Greenhouse Geisser correction]. Pairwise comparisons of the Set levels showed that the response times in the Fullset condition were significantly slower than in the Subset condition [*F*(1, 41) = 11.725, Bonferroni corrected *p* = 0.012, part. $\eta_{p}^{2}=0.456$, Δ_*Full,Sub*_ = 89.103 ms]. The other contrasts were not significant.

### 3.5. EEG Results

**Figure 6:**
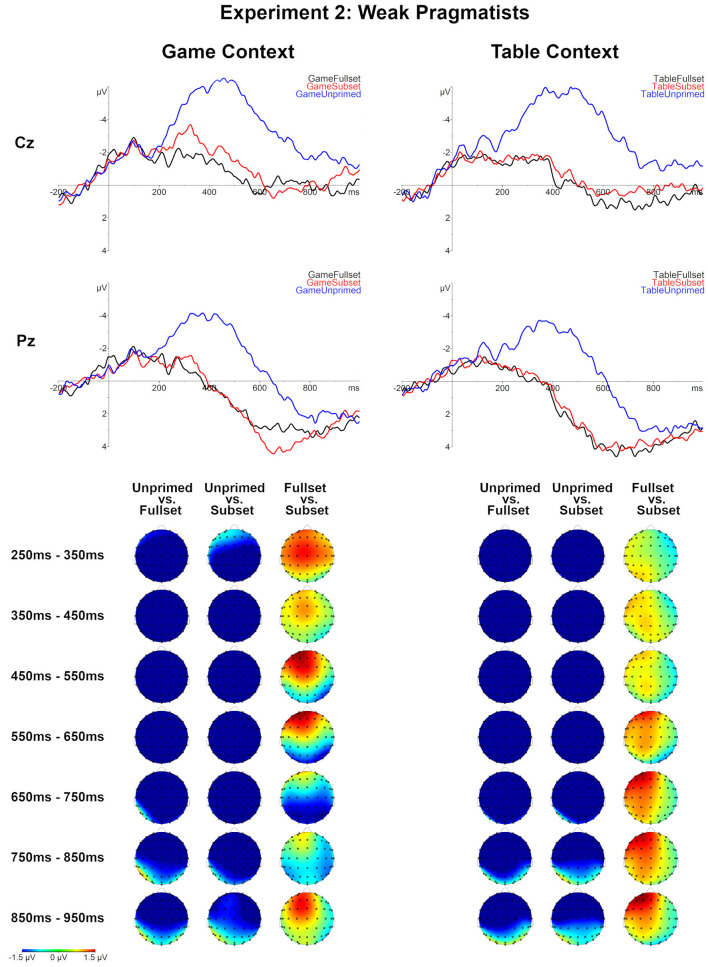
Grand averages for all conditions for weak pragmatists (*N* = 15), Experiment 2. Topographical maps of the differences between the compared Set levels separately for the Table and Game context in consecutive 100 ms time-windows, between 250 and 950 ms post-onset.

The ERPs were analyzed for 15 weak pragmatists. Similar as in Experiment 1, the Unprimed conditions showed a larger negativity effect relative to Subset and Fullset conditions for both context types (Table 5 and Figure 6). The negativity started around 200 ms post-onset and lasted almost till the end of the epoch. Unlike in Experiment 1, the comparison between Table-Fullset and Table-Subset did not show any significant effect. However, similar to the Experiment 1, the comparison between Game-Fullset and Game-Subset showed a late posterior negativity effect, which was marginally significant (the corresponding cluster latency and significance: 596–736 ms, *p* = 0.069). In addition, the comparison between Game-Fullset and Table-Fullest also showed a posterior negativity effect (612–804 ms, *p* = 0.0074).

**Figure 7:**
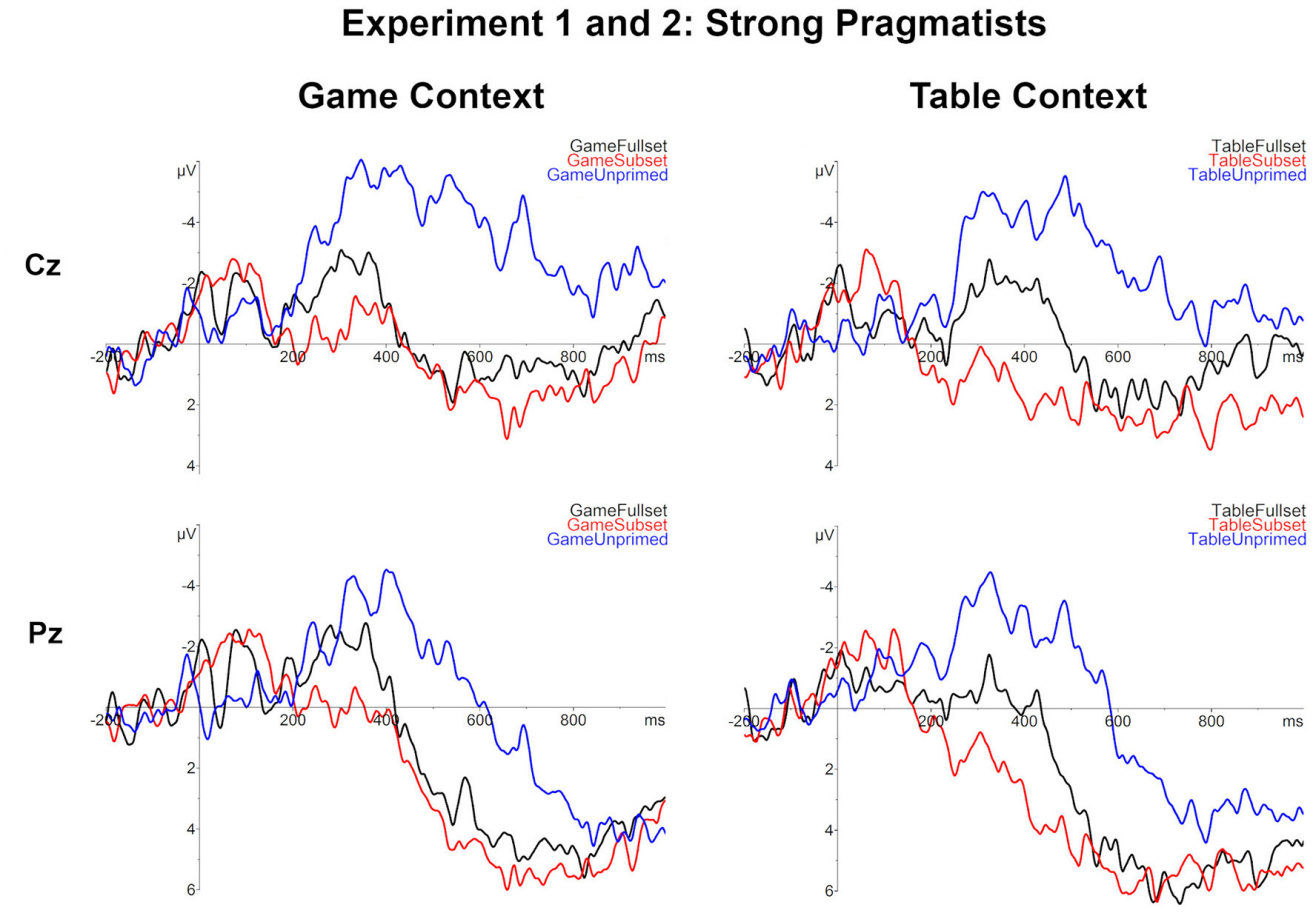
The comparison of subject averages in all conditions for the strong pragmatists, including the subject who displayed a mixed weak/strong response pragmatic response pattern (*N* = 4).

## 4. Discussion

In this paper we present the first ERP experiments testing the online processing of sentences with the weak scalar *some* in contexts where the speaker competence assumption does not hold. On the behavioral level we tested how the epistemic access to the quantified domain modulates the interpretation of the quantifier. By measuring the response times and ERPs we further tested how the online predictive processes are modulated by the quantifier interpretation on the one hand and the epistemic access to the domain on the other hand.

### 4.1. Appropriateness Judgments: Interpreting Truth and Appropriateness

Perhaps the most striking result at the behavioral level is a low ratio of pragmatic responses in the current experiments. In Experiment 1, only about 1/4 of the participants responded pragmatically, i.e., evaluated the target utterances with *some* as not appropriate in the Table-Fullset condition. Notably, practically all of these pragmatic responders accepted *some* as appropriate in the Game-Fullset condition, which means that they derived only the primary implicature. The strong pragmatic reading was adopted by only one participant in Experiment 1, and three more in Experiment 2, who judged the target *some*-utterances as inappropriate not just in the Table-Fullset but also in the Game-Fullset condition. Although the strong pragmatic interpretation is inconsistent with the neogricean view but predicted by the grammatical theory, it is not clear whether one can take such isolated responses as conclusive evidence to decide between the two approaches. Idiosyncratic response patterns may be observed due to various factors, for instance, as a strategic choice of an individual.

For Experiment 2, we only selected those participants who, based on a short questionnaire, were likely to display a pragmatic response pattern. In spite of that, almost one third of the tested individuals still responded logically during the actual EEG experiment. This result indicates that some features of the design made our participants more prone toward the logical interpretation, perhaps suppressing the implicature that they initially considered.

The low proportion of pragmatic relative to logical responses stays in contrast to the usual outcome of studies on scalar implicatures. For instance, Spychalska et al. (2016) reported an almost 50/50 divide between *pragmatists* and *logicians*. The reasons why so few subjects derived scalar implicatures in the current study may be manifold, including the presence of the partial access scenarios in the design, the proportion of other quantifiers used in the experiment, the modality of the stimuli presentation, as well as the nature of the task itself. Based on the oral feedback given by many of our participants, they found the question of whether the sentence is appropriate as more, rather than less, tolerant than the question about the sentence's truth-value. Some of the logical responders spontaneously and explicitly said that, strictly speaking, *Some of the cards on the table contain Xs* is not true when there are Xs on all of the cards; yet, in spite of not being true this sentence was still, according to them, “kind of appropriate.” From a philosophical perspective such an interpretation is puzzling, since pragmatically infelicitous statements are on theoretical grounds considered semantically true but somewhat “not good enough.” However, pre-theoretical intuitions of ordinary language users regarding truth may be dramatically different from the notions used by philosophers or semanticists: It seems that according to this alternative view, a sentence might not be considered true, but still “good enough.” In other words, the fact that a sentence was “false” seemed, for some subjects, not sufficient to judge it as necessarily inappropriate. At the same time, sentences that were known to be *semantically false* were almost unanimously judged as inappropriate, except for the occasional misinterpretation cases, that we discuss below.

Epistemic uncertainty seemed to also play a significant role for the response patterns. Some subjects struggled with the intended interpretation of the task, namely, they accepted the target utterances with *some* as appropriate in the Game-Unprimed condition. After the experiment they explained that they were not willing to judge a speaker's statement as not appropriate if they themselves could not know the truth-value. Such an interpretation of the Game-Unprimed condition was surprising since all of our subjects seemed to have understood the task when given the instructions and they responded correctly during the exercise session. They were explicitly told that an utterance can only be regarded as appropriate if the *speaker knows* that it is true. However, this instruction did not seem to be sufficiently internalized. For some of the participants it was still not acceptable to “tell Lena” that she is wrong if they could not know it themselves. Other participants occasionally expressed doubt whether Lena had some hidden knowledge about the closed cards, for instance, she might have cheated. The reasons for such interpretations could be manifold, e.g., the former could be due to politeness or face-saving strategies (Brown and Levinson, 1987), the latter could be a case of epistemic vigilance as discussed by Sperber et al. (2010). In general, it appears that the inability to know the sentence's truth-value made some subjects more careful in rejecting the statement as inappropriate. This confusion in the task interpretation also suggests that a setting, where the epistemic access to the scenario is manipulated, requires more complex reasoning than a simple sentence-picture verification paradigm does.

The fact that our participants lacked any privileged access to the scenario might also explain the discrepancy between our results and those by Dieuleveut et al. (2019), who report a significant rate of strong pragmatic readings. Unlike in our experiment, their participants always had access to the whole information and, thus, they always knew whether all cards are hearts in the real world. This factor might have endorsed the egocentric perspective in interpreting pragmatically ambiguous statements, resulting in a higher rate of strong pragmatic readings. Moreover, their trials were constructed in such a way that the ignorant speaker was always contrasted with the knowledgeable one. This contrast might have also biased the subjects toward more accurate statements over pragmatically ambiguous ones (“Mary can't say that but Peter can”). Another important difference between their design and ours is the type of question used: *Can she say that?* vs. *Is it appropriate?* However, the instructions provided by Dieuleveut et al. (2019) and especially the feedback given to the exercises is very similar to ours, i.e., in their study it is explained that Mary (or John) can say a statement if they are sure that it is true, whereas the reasons for a negative response could be either that a statement is known to be false or that the speaker does not have enough information to know whether it is true or false. We introduced the same limitation to the interpretation of “appropriate” by instructing the subjects that statements were appropriate if the speaker knew that they were true, and inappropriate if they were visibly false or if the speaker lacked sufficient evidence. Thus, in principle, the indented interpretation of the question was very similar across the two studies, although one cannot exclude that a different formulation had an effect on the divergent response patterns.

### 4.2. Reaction Times Reveal a Processing Cost of the Primary Implicature as Well as a Cost of Processing Partial Access Scenarios

Multiple prior studies have demonstrated that SIs are computed at a significant cost, resulting in longer response times for the pragmatic relative to logical interpretation of *some*. In line with these results, in Experiment 1 we also observed that the weak pragmatic interpretation led to a significant delay in responses for the Table-Fullset condition. Given that the pragmatic responses in the Table-Fullset condition were also slower than the “inappropriate” judgments in the Table-Unprimed condition, this effect cannot be simply attributed to the type of judgment (“inappropriate”) and may indicate a processing cost in deriving the primary implicature.

In addition, in Experiment 1, both the Game-Fullset and Game-Unprimed conditions showed slower responses relative to the Game-Subset condition. In the Game-Unprimed condition these slower responses could, in principle, be explained by assuming that the “inappropriate” judgments are more effortful than the “appropriate” ones. However, the response times in the Game-Unprimed condition proved not only longer relative to the other Game conditions but also relative to the Table-Unprimed condition, where the same type of behavioral judgments (“inappropriate”) were given. As in the Table-Unprimed condition the target utterances were visibly false, whereas in the Game-Unprimed condition their truth-value was unknown, the significantly slower responses in the latter relative to the former condition seem to be driven by the increased costs related to the processing of the unknown semantic status of an utterance. By contrast, in the Game-Fullset condition both logicians and weak pragmatists gave “appropriate” judgments. Thus, the longer response times in this case relative to the Game-Subset condition can be explained in terms of some level of epistemic uncertainty related to the partial access scenario.

These response time results partially replicate in Experiment 2. In this case, only weak pragmatists are included in the analysis and we observe slower responses in the Fullset relative to Subset condition in both contexts. This supports the prior finding that the Fullset condition, in which the implicature has to be considered (and is derived in the full access but inhibited in the partial access context), leads to a delay in sentence evaluation.

### 4.3. ERP Results: Priming-Related Effect

The N400 effect observed for the Unprimed conditions relative to the Subset and Fullset conditions, for both context types, is consistent with the literature that predicts the modulation of the N400 by priming. In our study, the critical words in the Unprimed conditions were not presented in the respective scenarios, unlike in the Subset or Fullset conditions. As primed words tend to trigger smaller N400 ERPs relative to unprimed words, it is unsurprising that the Unprimed conditions showed an N400 effect relative to the other conditions. What is less expected is the lack of any ERP effect between the Game- and Table-Unprimed conditions, especially in the light of longer response time found for the former relative to the latter. One possibility is that the effect related to epistemic reasoning does not show in the early processing of naturally spoken sentences, and occurs at a later stage, when the behavioral response is given.

### 4.4. ERP Results: Shallow Processing of the Primary Implicature

The comparison between Table-Fullset and Table-Subset conditions allowed us to test the implicature processing in the full access context. Let us recall that in the study by Spychalska et al. (2016), in a similar comparison, i.e., between *Some-infelicitous* and *Some-True* conditions, pragmatists showed a biphasic N400/P600 effect, whereas no effect was observed for logicians. In the current study, similar to the prior one, no effect was observed for logicians. Although, in Experiment 1, weak pragmatists showed a late posterior negativity effect for the Table-Fullset condition, starting after 600 ms post-stimulus onset, this effect did not replicate in Experiment 2. This result indicates that implicature was processed shallowly and with delay. The signature of the cost related to deriving the primary implicature could only be observed in longer response times in the Table-Fullset condition recorded for weak pragmatists.

### 4.5. ERP Results: The Cost of the Epistemic Step and the Role of Alternative Contexts

To test the effect of epistemic access for the processing of pragmatically ambiguous sentences with *some*, we compared Game-Fullset with Game-Subset conditions, as well as each of the Set conditions across the context type (Game vs. Table). We expected that the observed effects should be modulated by the quantifier reading, i.e., by the appropriateness judgments in the Table-Fullset and Game-Fullset conditions. For instance, in the case of strong pragmatic interpretation, an N400 effect was expected for the Game-Fullset relative to Game-Subset context. Unfortunately, as only three participants consistently adopted this reading (one in Experiment 1 and two in Experiment 2) and one subject in Experiment 2 partially adopted this reading, no statistical evaluation of this hypothesis was possible^8^.

For weak pragmatists, the comparison between Game-Fullset and Game-Subset conditions showed a late, sustained negativity effect. This effect was significant in Experiment 1 and marginally significant in Experiment 2. The Game-Fullset condition also showed a late negativity effect relative to the Table-Fullset condition, which was marginally significant in Experiment 1 and significant in Experiment 2. It is important to note that there were no differences between the two experiments other than the method of subjects' selection: For the second experiment, the participants were pre-selected based on a short questionnaire that included one task corresponding to the Table-Fullset condition. This procedure was intended to reduce the number of unnecessary recordings (i.e., disregard those subjects who were likely to display a logical response pattern). Thus, the participants tested in Experiment 2 had seen a few questions similar to those used in the EEG experiment already before coming to the lab. Although it cannot be ruled out, it is unlikely that this pre-exposure to the experimental task would have had a significant effect on the observed ERP patterns. However, due to the interruption of the testing caused by the pandemic outbreak we were able to record only 15 consistent weak pragmatists, which is also suboptimal from the point of view of statistical power. Given that the reported effects showed a consistent trend across experiments both with respect to the topography and latency, they are likely to reflect real effects and the marginally significant *p*-values are due to small sample sizes.

Late sustained negativities of posterior distribution were observed earlier in various linguistic contexts. For instance, Politzer-Ahles et al. (2012) observed an effect of this sort in response to pragmatically infelicitous quantifiers. In this study, sentences such as *In this picture, some of the girls are sitting on the blankets sunbathing* were read as descriptions of pictures that matched (with a subset of the girls on the blankets) or mismatched the interpretation with the scalar implicature (with all of the girls on the blankets). The ERPs measured at the onset of sentence-initial quantifiers showed a posterior negativity effect after 500 ms post-stimulus onset for the mismatching relative to the matching scenario. This effect was interpreted as a signature of “effortful pragmatic reanalysis," which the authors further suggested specifically involved “inhibiting the pragmatic reading of *some* of and retrieving the semantic reading.” The effect was observed for the whole tested population without differentiating between pragmatic and logical responders. Acceptability judgments that would allow to distinguish between the pragmatic and logical interpretation followed only 6 trials per condition. Given that most of the participants were considered to be inconsistent responders, no between-group analysis was conducted.

Late posterior negativity (LPN) effects have also been observed in memory research: A large number of ERP studies on recognition memory observed that responses for old relative to new, correctly classified items, tend to trigger a sustained negativity effect over posterior sites beginning at approximately 600 or 800 ms post-onset and lasting till the end of the 1,000 ms or even 2,000 ms long epoch (for a review see Mecklinger et al., 2016). However, due to heterogeneity of experimental manipulations giving rise to this effect, it has been suggested that the LPN may not reflect a single process and is unlikely to constitute a direct correlate of episodic recollection, especially that this effect was shown to be sensitive not just to episodic but also to semantic memory tasks, that require more specific reconstructive processes (cf. Johansson and Mecklinger, 2003; Herron, 2007). In attempts to describe the functional sensitivity of LPN, many authors have linked it, inter alia, to late/extended retrieval processes (Dzulkifli and Wilding, 2005; Bergström et al., 2013), post-retrieval maintenance/evaluation of contextual information (Johnson et al., 2008), contextual familiarity (Addante et al., 2012), context monitoring and contextual retrieval of task relevant attributes (Goffaux et al., 2008), or enhanced need to monitor response conflict between suppression and automatic retrieval (Hu et al., 2015). As a common denominator of these proposal, the LPN seems to be related to late processes that have to do with (re)evaluation/monitoring of the context. Assuming a functional similarity between the late posterior negativity observed in the current experiments for weak pragmatists and the memory-related LPN, one could argue that in our studies this effect arises as a result of extended and possibly inference-driven context monitoring processes that may be related to reevaluation of the scenario, in particular, reconsidering the speaker's epistemic access. As weak pragmatists are sensitive to the implicature in the full access context, one can hypothesize that for this group the partial access context engages processes related to the evaluation of speaker competence assumption, and eventually to implicature inhibition.

By contrast, for logicians a differential pattern of effects is observed, namely, a robust N400-like negativity for the Game-Subset relative to Game-Fullset condition, as well as relative to the Table-Subset condition. At first, this result may appear puzzling but it can be explained by taking into account global pragmatic effects arising from the competition between two alternative contexts contrasted in the experiment. Based on the monotonicity properties of *some*, in the Subset condition, the Game restriction is somewhat less informative than the Table restriction, and in addition the Game restriction is more informative when used in the Fullset rather than in the Subset condition. Accordingly, this informativity relation was also expected to modulate predictive processes during sentence comprehension, leading to larger N400 ERPs for the less informative utterances. The effects observed for logicians are precisely in the line with this prediction. Although for weak pragmatists the N400-like effect for the Game-Subset vs. Table-Subset condition was also observed in Experiment 1, Experiment 2 did not show a similar, even marginally significant, effect for this group. Thus, the N400-like negativity in the Game-Subset condition appears to be primarily modulated under the logical interpretation. Such an outcome is expected if one observes that, under the logical interpretation, *some* may be considered the most “optimal” quantifier (out of all contextually provided in the experiment) to use in cases where insufficient information is provided about the domain, which is exactly the Game-Fullset context. Thus, in spite of equivalent appropriateness judgements in all of the Subset and Fullset conditions, under the logical interpretation, *some cards in the game* may be primarily used as means of expressing uncertainty about the whole domain, and consequently perceived as less optimal in the Subset condition, where *some cards on the table* would be more informative. Most importantly, logicians are defined as those participants who, based on their appropriateness judgments, appear not to have derived the primary implicature. This does not mean that they are insensitive to pragmatic mechanisms as such. In this case, we observe that the global pragmatic effects, which are based on contextually provided alternatives, played a primary role in modulating their processing pattern.

### 4.6. Response Times and ERPs Reveal Processes at Different Stages

It is noteworthy that some of the contrasts that showed significant reaction time differences did not yield significant ERP effects. For instance, the weak pragmatic response was associated with longer response times in the Table-Fullset condition relative to Table-Subset, the late negativity ERP effect in this comparison was only observed in Experiment 1 and did not replicate in Experiment 2. This result suggests that the primary implicature processing did not occur incrementally but rather with delay, and only incurred cost at the stage of the behavioral judgment. In addition, the response times in condition Game-Unprimed turned out significantly longer than those in condition Table-Unprimed, no significant ERP effect is observed in this comparison, at least not in the analyzed epoch, i.e., up to the 1,000 ms post-stimulus onset. Given the natural and relatively fast pace of presenting the auditory stimuli, some of the processes related to epistemic reasoning might have been impossible to detect in the early time-window (up to 1,000 ms), when the ERPs are measured. These processes, however, still left a mark in the responses times, as the responses were given at a later stage.

### 4.7. Differences Between the Current and the Prior ERP Study on Scalar Implicatures

The results observed in the current study differ to a large extent from those observed in Spychalska et al. (2016), including a different distribution of behavioral responses (a lower proportion of pragmatists in the current study) and a different pattern of ERP results in the comparable conditions. The most striking result is the lack of any robust effect for weak pragmatists in the Table-Fullset vs. Table-Subset comparison. Let us recall that in a similar comparison in Spychalska et al. (2016) pragmatists had a combination of the N400 and the P600 effect. These differences in results may be linked to some important aspects of both designs, including the distribution of filler trials, the modality of the stimuli presentation (visual vs. auditory sentence presentation), the type of task and, finally, the very presence of the partial access scenarios in the current experimental setting.

First, although the current study used filler trials of a similar sort as the prior one, namely, trials with *all* and *no* as well as with numerals, the probability of encountering such items was lower compared to the prior study. In Spychalska et al. (2016), there was an equal number of trials with *all* and *some*, each of which constituted 40% of all trials, whereas the remaining 20% were fillers with such quantifiers as *no, most*, and with bare numerals. In the current study, due to the fact that we needed as many as six different conditions with *some*, as well as additional fillers with *some*, trials with *some* altogether constituted approximately 66% of all trials, whereas those with *all* only about 10% (10% of all trials were those with *no* and 14% were other fillers). Prior studies have shown that the type and proportion of filler items may have a significant effect on the time-course of scalar implicature processing: For instance, Dieussaert et al. (2011) showed that participants tended to be less consistent in the chosen logical or pragmatic interpretation if the filler ratio was higher. Degen and Tanenhaus (2015) showed that implicatures were processed more costly if other scalar terms such as number words were available in the context. Finally, Augurzky et al. (2019) observed that the contrast between *all* vs. *some* may prime the scalar implicature, more specifically, if such a contrast was not present in the context, the implicature was processed more shallowly. This last result appears to be particularly relevant for our study, namely, the lower proportion of trials with *all* in the current design could have contributed to some extent to the observed lower proportion of pragmatic responders as well as to the more shallow processing of the implicature. Still, the contrast with *all* was not absent in the design and the difference was only in the probability of such items. To evaluate the role of this factor we can refer to the study by Hunt et al. (2013), which used a very similar design to Spychalska et al. (2016), but a lower proportion of *all* vs. *some* trials: In this study there were 171 target trials with *some* (divided into three conditions: *true/false/infelicitous*) and additional 171 filler trials that were distributed between *all, no* and *some*. Although the precise proportion is not reported, it is clear that the probability of trials with *all* was much lower than those with *some*^9^. In spite of having a lower ratio of *all* vs. *some* items, Hunt et al. (2013) observed a very similar pattern of results as Spychalska et al. (2016), namely, a biphasic N400/P600 effect for the *infelicitous* relative to the *true* condition, that was only apparent for the pragmatic responders. Thus, although a diminution of the expected N400/P600 effect relative to the probability of *all* vs. *some* items seems plausible, given that the *all* items were still contextually active in the current experiment, the filler distribution is unlikely to explain the complete lack of the expected N400/P600 effect.

Second, as discussed earlier, the different form of the judgment task, namely, appropriateness rather than truth-value judgements, appears to have affected the interpretation of the utterances resulting in a lower proportion of pragmatic responders. This is evident from the oral feedback provided by the participants as well as from some of the misinterpretation cases of the partial access conditions. It is, however, unlikely that the effect of the question type reached beyond the distribution of the behavioral responses and also modulated the time-course of the implicature processing. Although the P600 effect is considered task-dependent, the N400 effect is generally taken to occur independently of the task. Thus, the different type of task should not prohibit the N400 from occurring in response to the condition inconsistent with the implicature.

The third important aspect is the modality in which the linguistic stimuli were presented, which also determined the time-course of the stimuli presentation. It is unlikely that the auditory vs. visual presentation of the sentence made a significant difference to the general pattern of the effects, since language-related ERP components, such as the N400 and the P600 are generally modality-independent (Osterhout and Holcomb, 1993; Kutas and Federmeier, 2011). Auditorily presented sentences tend to trigger P600 effects in response to syntactic violations similar to visually presented ones (Osterhout and Holcomb, 1993). N400 effects were also observed both for visual and auditory words, although some cross-modality differences in the time-course and topography of these effects have been observed: Auditory N400s tend to begin earlier, last longer, and have a slightly more frontal and less right-hemisphere biased topography (Kutas and Van Petten, 1994; Kutas and Federmeier, 2011). Different patterns of priming-driven N400 effects between auditory and visual words have been reported by Holcomb et al. (1992) and Holcomb and Anderson (1993), who argued for a modality-specific modulation of the semantic processing system. Yet, these modality specific differences seem to concern only minor spatio-temporal variation in the component's characteristics, rather than the occurrence of the effects under similar experimental manipulations. Nevertheless, the auditory modality had also consequences for the time-course and pace of presenting the linguistic stimulus. In the study by Spychalska et al. (2016), the quantifying phrase was presented before the pictures, which means that during the inspection of the scenario the subjects could have already computed the implicature and shape their expectations regarding the sentence-final noun. From the quantifier onset until the critical word onset there were in total 5,000 ms. In the current study, the sentence was heard during the inspection of the scenario: The average sentence duration was approximately 2, 502*ms* (2,702 ms was the average length of the audio file, which includes the approximate 200 ms of the pre-stimulus silence). From the onset of the sentence until the onset of critical word there were on average about 1,939 ms (based on the 2,139 average onset of the critical noun minus the 200 ms silence onset), whereas from the onset of the scenario until the onset of the critical noun there were on average 3,939 ms. If the implicature calculation is indeed an effortful process, then it is possible that in the current study there was not enough time for the implicature to be computed and, thus, it could not modulate the predictions for sentence-final words. To discuss the role of this factor, we can again refer to the study by Hunt et al. (2013) for a comparison. In this experiment, the scenario was presented first and consisted of two screens: the first screen with a set of items of various sorts, e.g., 4 steaks, 4 apples and 4 brownies, and the second screen, where some items from one category (e.g., steaks) and all items from another category (e.g., apples) were cut. Sentences of the form *The student has cut some of the* apples *in this story* were presented word-by-word after the scenario, using rapid serial visual presentation with 300 ms for each word and 200 ms between words. Thus, from the onset of the quantifier until the onset of the critical noun there was only a time span of about 1,500 ms. This would suggest that the N400 effect in response to the scenario-based implicature mismatch may also be observed for stimuli presented in a natural pace. Yet, one must also take into account that in Hunt et al. (2013) the scenario preceding the sentence presentation was shown for a total of 13,000 ms (the duration of the two screens was 7,500 and 5,500 ms respectively), i.e., much longer than in our current experiment. Given that in Hunt et al. (2013) the majority of trials used the quantifier *some*, and the set of all other quantifiers used was limited to *some*/*all*/*no*, one cannot exclude that some strategic anticipation of the nouns' match/mismatch in relation to each of the potential quantifiers happened already during the scenario inspection to facilitate the processing of the upcoming sentence.

The last factor to be considered is the presence of the partial access scenarios. This aspect of the design distinguishes the current study both from the study by Spychalska et al. (2016) and the one by Hunt et al. (2013). Thus, it is a likely candidate to explain the discrepancy between the current results and the prior ones. The competition between the two alternative contexts and the presence of the closed cards in the scenarios possibly triggered processes related to the reevaluation of the speaker epistemic access. This epistemic component led to non-incremental implicature processing: Primary implicatures were derived with delay and post-propositionally, which explains the absence of any clear ERP effect for weak pragmatists in the critical Table-Fullset vs. Table-Subset comparison. The LPN effect observed for this group in the partial access context (Game-Fullset vs. Subset) may indicate engagement of processes related to the increased context evaluation/monitoring. This effect may be explained by the hypothesis that weak pragmatists perform epistemic reasoning related to the evaluation of the competence assumption, which leads then to implicature inhibition in the partial access context. In the studies by Hunt et al. (2013) or Spychalska et al. (2016), where only full access scenarios were presented, such epistemic processes were not contextually induced. By contrast, the processing patterns of logicians appear to be modulated rather by global pragmatic mechanisms related to informativeness of each of the alternative quantifying expressions (*some cards on the table* vs. *some cards in the game*) as applied to the Fullset or Subset condition. Since, in this particular setting, *some cards in the game* is semantically weaker than *some cards on the table*, the use of the former expression may be considered less informative in the Subset condition. This effect is further strengthened if the logical reading is adopted, since in this case, *some* can be taken as the most optimal means of expressing uncertainty about the whole domain, resulting in the *some cards in the game* quantifying phrase being the most optimal one in the Fullset scenario.

### 4.8. Conclusion

Prior studies on the role of speaker competence assumptions in deriving scalar implicatures have been rather scarce and up to date there have been no ERP studies investigating the real-time processing of scalar implicatures in partial access contexts, i.e., contexts where the speaker's competence cannot be assumed. In this paper we present both behavioral and ERP data to fill this gap.

First, we observe a very low percentage of pragmatic responses in the full access contexts, where the speaker competence assumption holds. Thus, primary scalar implicatures were derived less frequently than in other experiments reported in the literature. Moreover, almost all those subjects who did derive the primary implicature did not derive the secondary implicature in the partial access context: Only three subjects in total applied the strong pragmatic interpretation (four if we include the one additional subject who was switching between the weak and strong interpretation). This result is striking as it indicates, on the one hand, that the strong pragmatic interpretation appears at best as an isolated response pattern, and on the other hand, that the presence of partial access contexts in the design suppresses the pragmatic interpretation as such. This effect may be also due to the type of judgment task used in our study: Appropriateness judgments may have been interpreted less rigidly than truth-value judgments which led some subjects to treat sentences as appropriate in spite of the fact that they would not have evaluated them as true in the strict sense. It is also interesting that partial access contexts showed problematic also in other cases: In the Game-Unprimed condition, where the sentence semantic status is unknown, less accurate judgments were observed leading to a number of systematic misinterpretation cases. These results also show that speaker epistemic status manipulations are experimentally problematic, possibly since they require from the participants to perform belief reasoning and to represent the presumed belief state of the virtual agent. This may lead to a number of errors both in the sense of varied interpretations of what the indented (in the experiment) agent's belief state is as well as errors of misidentification of one's own belief with that of the agent.

As the most important result we showed that partial access contexts involve a processing cost, which left mark both in the accuracy, response times and elicited ERPs. The condition Game-Unprimed, which involves the highest level of epistemic uncertainty level, was associated not only with the highest level of response errors but also with longer response times both relative to Game-Subset and Game-Fullest conditions, as well as relative to the Table-Unprimed condition. This result was comparable for both logicians and weak pragmatists. Although no ERP effect was observed when comparing the Table- and Game-Unprimed conditions, the difference in accuracy and response times is interpreted as evidence of increased cognitive demands related to epistemic uncertainty in the partial access context. Furthermore, our results suggest that, in the current experimental setting, deriving implicatures was cognitively costly: Weak pragmatist responded slower in the Table-Fullset condition relative to other conditions but showed no robust ERP signature of implicature processing. This indicates that deriving the primary implicature occurred not incrementally and late. Longer response times are also observed for weak pragmatists in the Game-Fullset relative to the Game-Subset condition. In addition, the Game-Fullset condition shows a late posterior negativity effect relative to the Game-Subset and Table-Fullset conditions, which we interpret as a signature of epistemic context-reevaluation that led to implicature inhibition. Due to the small number of weak pragmatists in both experiments, some of these effects are marginally significant and, hence, should be treated with caution. However, as we can see a consistent trend between the two experiments, the observed patterns are likely to reveal real effects. We argue that the observed processing patterns are inherently related to the contrast between the partial and full access contexts present in the experiment. In the case of weak pragmatists, who are sensitive to the implicature at the quantifier level, this contrast leads to non-incremental implicature processing and epistemic context-reevaluation. For logicians, who do not derive the primary implicature, the processing patterns are primarily modulated by the informativity relation between the two domain restrictions.

To sum up, our experiment shows that if the general context raises the question of whether or not the speaker has sufficient information to make the statement, the implicatures are processed as postpropositional inferences rather than as automatic and incremental. Thus, the contrast between full and partial access contexts seems to enforce on the listeners, at least on those who choose the pragmatic interpretation, taking the “epistemic step”: reconsidering whether or not the speaker is epistemically competent. Although, our conclusions are not claimed to provide any definite answer in the debate, our results appear more in line with the traditional, pragmatic account rather than with the grammatical view on scalar implicatures.

## Data Availability Statement

The raw data supporting the conclusions of this article will be made available by the authors, without undue reservation.

## Ethics Statement

Ethical review and approval was not required for the study on human participants in accordance with the local legislation and institutional requirements. The patients/participants provided their written informed consent to participate in this study.

## Author Contributions

MS designed and programmed the experiment, supervised the stimuli preparation and the data collection, pre-processed and analyzed the data, and wrote the manuscript. LR contributed to the design, prepared the visual and auditory stimuli, collected the data, and contributed to the manuscript preparation. PS provided the infrastructure for the auditory stimuli recording, contributed to the design, and results interpretation. MW provided the infrastructure and funding for the EEG recording, contributed to the design, and results interpretation. All authors contributed to the article and approved the submitted version.

## Conflict of Interest

The authors declare that the research was conducted in the absence of any commercial or financial relationships that could be construed as a potential conflict of interest.

## Footnotes

^1^By using “appropriate/inappropriate” rather than “can/cannot say that” we avoided the reading of “is able to,” making it more natural for the subjects to judge a past event (i.e., Lena's utterance that has already happened). Another benefit is that “appropriate” is used less frequently than “can” in German and, thus, it is easier to establish a certain, intended reading of “appropriate” in an experimental setting, avoiding any potential “fuzzy” readings that might occur with the use of “can”.

^2^See Supplementary Materials for the full text of the instructions.

^3^The frequency *value* *v* of a word *w* is equal to *log*_2_ of the quotient of the frequency of the word “der” and the frequency of the word *w* in the corpus. The values were checked with the *Wortschatz Leipzig*.

^4^See Supplementary Materials.

^5^The statistical methods were selected to correspond to those from Spychalska et al. (2016) for a better comparability of the results.

^6^No outliers shorter than the mean −2 SD were found.

^7^Although every participant who pre-screened as pragmatist was offered a date, not all wanted to come for the EEG experiment; in addition, some of the scheduled dates had to be canceled due to the covid-19 pandemic outbreak.

^8^Note that based on the visual inspection, the ERPs observed for these participants are consistent with this hypothesis (see Figure 7).

^9^Assuming the likely even distribution of *all*/*some*/*no* across the filler trials, the proportion of *all* would be ca. 16% and the proportion of *some* approximately 66%.
