# Supplementary material for: The Cost of the Epistemic Step: Investigating Scalar Implicatures in Full and Partial Information Contexts
Source: Front Psychol. 2021 Jul 19;12:679491. doi: 10.3389/fpsyg.2021.679491 (PMC8328393; doi:10.3389/fpsyg.2021.679491)
Supplement: Supplementary file 1 [file Data_Sheet_1.pdf]

Supplementary Materials for the manuscript *The cost of the epistemic step: Investigating scalar implicatures in full and partial information contexts*

Maria Spychalska, Ludmila Reimer, Petra B. Schumacher, and Markus Werning

May 19, 2021

Table 1: A detailed list of all filler trials: for each type and subtype of filler, a number of trials is given.

Quantifier		Number of object types shown		Is the critical word shown		Scene: two cards outside the table		Truth Value		Context type	
Einige	40	one	16	yes	8	face down	4	true	4	table	2
										game	2
						not dealt	2	true	2	game	1
										table	1
						face up	2	true	2	table	1
										game	1
				no	8	face down	4	unknown	4	table	2
										game	2
						not dealt	2	false	2	game	1
										table	1
						face up	2	false	2	game	1
										table	1
		two	8	yes	4	not dealt	2	true	2	table	1
										game	1
						face up	2	true	2	table	1
										game	1
				no	4	not dealt	2	false	2	table	1
										game	1
						face up	2	false	2	table	1
										game	1
		three	16	yes	16	face down	8	true	8	game	4
										table	4
						not dealt	4	true	4	game	2
										table	2
						face up	4	true	4	game	2
										table	2
Alle	40	one	12	yes	8	down	4	false	2	game	1
										table	1
								true	1	table	1
								unknown	1	game	1
						face up	2	false	1	table	1
								true	1	game	1
						not dealt	2	false	1	game	1
								true	1	table	1
				no	4	face down	2	false	2	game	1
										table	1
						face up	1	false	1	game	1
						not dealt	1	false	1	table	1
		two	18	yes	12	face down	6	false	3	table	1
										game	2
								true	2	table	2
								unknown	1	game	1
						face up	3	false	1	table	1
								true	2	table	1
										game	1
						not dealt	3	true	1	game	1
								false	2	table	1
										game	1
				no	6	two cards face down	3	false	3	game	1
										table	2
						face up	1	false	1	game	1
						not dealt	2	false	2	table	1
										game	1
		three	10	yes	10	face down	5	false	3	table	1
										game	2
								true	1	table	1
								unknown	1	game	1
						face up	3	false	1	table	1
								true	2	game	1
										table	1
						not dealt	2	false	1	game	1
								true	1	table	1
Keine	40	one	16	yes	6	face down	3	false	3	table	2
										game	1
						face up	2	false	2	game	1
										table	1
						not dealt	1	false	1	game	1
				no	10	face down	5	true	2	table	2
								unknown	3	game	3
						face up	2	true	2	game	1
										table	1
						not dealt	3	true	3	game	1
										table	2
		two	16	yes	8	face down	4	false	4	game	2
										table	2
						face up	2	false	2	game	1
										table	1
						not dealt	2	false	2	game	1
										table	1

				no	8	face down	4	true	1	table	1
								unknown	3	table	1
										game	2
						face up	2	true	2	game	1
										table	1
						not dealt	2	true	2	game	1
										table	1
		three	8	yes	8	face down	4	false	4	game	2
										table	2
						face up	2	false	2	table	1
										game	1
						not dealt	2	false	2	table	1
										game	1
Weniger als drei/vier	20	one	8	yes	4	face down	2	false	2	table	1
										game	1
						face up	2	true	2	table	1
										game	1
				no	4	face down	2	true	1	table	1
								unknown	1	game	1
						face up	1		1	table	1
						not dealt	1		1	game	1
		two	8	no	4	face down	2	true	1	table	1
								unknown	1	game	1
						face up	1	true	1	game	1
						not dealt	1	true	1	table	1
				yes	4	face down	2	false	1	table	1
								unknown	1	game	1
						face up	1	true	1	table	1
						not dealt	1	true	1	game	1
		three	4	yes	4	face down	2	false	1	table	1
								unknown	1	game	1
						not dealt	2	true	2	game	1
										table	1
Mehr als zwei/drei	20	one	8	no	4	face down	2	false	2	game	1
										table	1
						face up	1	false	1	table	1
						not dealt	1	false	1	game	1
				yes	4	face down	2	true	1	table	1
								unknown	1	table	1
						face up	1	true	1	game	1
						not dealt	1	false	1	table	1
		two	8	no	4	face down	2	false	2	table	1
										game	1
						face up	1	true	1	game	1
						not dealt	1	true	1	table	1
				yes	4	face down	2	false	1	table	1
								unknown	1	game	1
						face up	1	true	1	table	1
						not dealt	1	true	1	game	1
		three	4	yes	4	face down	2	true	1	table	1
								unknown	1	game	1
						face up	1	true	1	game	1
				no		not dealt	1	false	1	table	1
Zwei/Drei	20	one	8	no	4	face down	2	false	1	table	1
								unknown	1	game	1
						face up	1	false	1	game	1
						not dealt	1	false	1	table	1
				yes	4	face down	2	false	1	table	1
								true	1	game	1
						face up	1	true	1	table	1
						not dealt	1	true	1	game	1
		two	8	no	4	face down	2	false	1	table	1
								unknown	1	game	1
						face up	1	false	1	table	1
						not dealt	1	false	1	game	1
				yes	4	face down	2	false	1	table	1
								unknown	1	game	1
						face up	1	true	1	game	1
						not dealt	1	true	1	table	1
		three	4	yes	4	face down	2	true	1	table	1
								unknown	1	game	1
						face up	1	true	1	table	1
						not dealt	1	true	1	game	1

Table 2: The overview of fillers: the table provides a sum of different categories of filler trials. Note: for each category, the sum of examples equals 180.

Category	*Subcategory*	*Number of examples*
Context	Table	90
	Game	90
Quantifier	Einige	40
	Alle	40
	Keine	40
	Weniger als drei/vier	20
	Mehr als zwei/drei	20
	Zwei/Drei	20
Number of object categories shown	one	68
	two	66
	three	46
Is the critical word shown in the scenario	yes	116
	no	64
Cards' configuration : two cards outside the table	Two hidden cards are dealt	86
	Only table cards dealt	47
	Two extra open cards are dealt	47
Truth-value	True sentences	78
	Known to be false sentences	78
	Unknown truth-value	24
Appropriateness	Appropriate sentences	74
	Inappropriate sentences	102
	Pragmatically inappropriate but logically appropriate sentences	13

Table 3: Exhaustive list of the exercise trials

Quantifier		Number of object types shown		Is the critical word shown		Scene: two cards outside the table		Truth Value		Context type	
Alle	2	two	2	yes	2	face down	2	true	1	table	1
								unknown	1	game	1
Keine	2	one	2	yes	1	not dealt	1	false	1	game	1
				yes	1	face up	1	true	1	table	1
Mehr als drei	1	two	1	no	1	not dealt	1	false	1	game	1
Mehr als zwei	1	three	1	yes	1	face up	1	true	1	table	1
Weniger als drei	1	one	1	yes	1	face up	1	true	1	table	1
Weniger als zwei	1	three	1	yes	1	face down	1	unknown	1	game	1

Table 4: The list of word combinations, with frequencies and English translations, used for the target trials. Note that the set of words in each column (word1, word2, word3) is the same, but the words are combined each time differently.

word1	translation	frequency	word2	translation	frequency	word3	translation	frequency
Bänke	benches	13	Kühe	cows	12	Gläser	glasses	12
Äpfel	apples	12	Igel	hedgehogs	13	Brücken	bridges	11
Enten	ducks	13	Gürtel	belts	12	Türen	doors	10
Monde	moons	13	Zebras	zebras	14	Eimer	buckets	13
Mäuse	mice	13	Socken	socks	12	Deckel	lids	13
Betten	beds	11	Tiger	tigers	11	Gurken	cucumbers	14
Rehe	deer	14	Leitern	ladders	14	Fäden	threads	12
Bären	bears	11	Sterne	stars	11	Enten	ducks	13
Möhren	carrots	14	Hüte	hats	14	Schlüssel	keys	11
Frösche	frogs	14	Kannen	jugs	16	Becher	mugs	13
Schlangen	snakes	12	Mützen	caps	14	Kissen	pillows	13
Hunde	dogs	10	Türen	doors	10	Knochen	bones	11
Hasen	bunnies	12	Gurken	cucumbers	14	Schweine	pigs	12
Birnen	pears	13	Torten	cakes	14	Mäuse	mice	13
Trauben	grapes	13	Kekse	cookies	14	Pfeifen	pipes	14
Schwäne	swans	14	Glocken	bells	12	Würfel	dice	13
Sessel	chairs	13	Geigen	violins	15	Stiere	bulls	15
Mäntel	coats	15	Haie	sharks	13	Pfannen	pans	15
Schleifen	bows	14	Lippen	lips	12	Hocker	stools	14
Körbe	baskets	14	Schirme	umbrellas	15	Mäntel	coats	15
Ziegel	bricks	14	Teddys	teddy bears	16	Schüsseln	keys	15
Häuser	houses	9	Bäume	trees	10	Messer	knives	10
Engel	angels	11	Affen	monkeys	12	Hosen	pants	12
Vögel	birds	11	Blumen	flowers	11	Lampen	lamps	12
Federn	feathers	12	Kerzen	candles	11	Schafe	sheep	12
Bücher	books	9	Uhren	clocks	12	Hühner	chicken	12
Mädchen	girls	8	Spiegel	mirrors	10	Augen	eyes	8
Schweine	pigs	12	Boote	boats	12	Ringe	rings	12
Hefte	books	14	Eulen	owls	14	Zähne	teeth	11
Koffer	suitcases	11	Beine	legs	10	Ruder	paddles	12
Taschen	bags	12	Würste	sausages	14	Biber	beavers	13
Hühner	chicken	12	Stühle	chairs	12	Duschen	showers	13
Lampen	lamps	12	Kissen	pillows	13	Nester	nests	15
Puppen	dolls	13	Drachen	kites	13	Bomben	bombs	12
Trommeln	drums	13	Schuhe	shoes	10	Beine	legs	10
Tassen	cups	13	Stifte	pencils	15	Puppen	dolls	13
Hämmer	hammers	17	Schwämme	sponges	16	Brote	loaves of bread	14
Gabeln	forks	16	Schaufeln	shovels	14	Flöten	flutes	15
Pilze	mushrooms	12	Knöpfe	buttons	14	Wölfe	wolves	12
Pinsel	brushes	13	Helme	helmets	14	Nüsse	nuts	13
Besen	brooms	13	Töpfe	pots	14	Palmen	palm trees	13
Autos	cars	8	Jungen	boys	9	Herzen	hearts	9
Vasen	vases	15	Muscheln	clams	13	Torten	cakes	14
Nadeln	needles	13	Ringe	rings	12	Bälle	balls	12
Schlüssel	keys	11	Münzen	coins	12	Fliegen	flies	12
Eier	eggs	11	Messer	knives	10	Hunde	dogs	10
Schals	scarves	14	Flöten	flutes	15	Münzen	coins	12
Brillen	glasses	13	Schnecken	snails	14	Kannen	jugs	16
Angeln	fishing rods	14	Ziegen	goats	13	Löffel	spoons	13
Hosen	pants	12	Ruder	paddles	12	Ziegen	goats	13
Fäden	threads	12	Gläser	glasses	12	Taschen	bags	12
Babys	babies	11	Flaschen	bottles	11	Kuchen	pies	10
Teller	dishes	11	Hemden	shirts	13	Bienen	bees	12
Sonnen	suns	15	Zäune	fences	13	Brillen	glasses	13
Schafe	sheep	12	Teufel	devils	11	Flaschen	bottles	11
Schränke	cupboards	14	Blätter	leaves	11	Tafeln	boards	12
Bögen	bows	14	Röcke	skirts	14	Waffeln	wafers	13

Jacken	jackets	13	Löffel	spoons	13	Kugeln	balls	12
Tische	tables	12	Löwen	lions	10	Kleider	dresses	12
Pfannen	pans	15	Sägen	saws	15	Kreuze	crosses	13
Nägel	nails	13	Ketten	chains	13	Tiger	tigers	11
Rosen	roses	12	Tüten	bags	13	Spinnen	spiders	13
Schwerter	swords	13	Kirschen	cherries	14	Räder	bikes	11
Kronen	crowns	11	Pferde	horses	11	Röcke	skirts	14
Käse	loaves of cheese	11	Augen	eyes	8	Schuhe	shoes	10
Herzen	hearts	9	Wolken	clouds	11	Reifen	tires	11
Pfeile	arrows	14	Kreuze	crosses	13	Zebras	zebras	14
Esel	donkeys	12	Zwiebeln	onions	13	Federn	feathers	12
Anker	anchors	13	Würfel	dice	13	Schlangen	snakes	12
Kämme	combs	17	Pflaumen	plums	15	Teddys	teddy bears	16
Pfeifen	pipes	14	Masken	masks	13	Kronen	crowns	11
Hörner	horns	14	Fässer	barrels	14	Gänse	geese	14
Raupen	caterpillars	15	Kegel	bowling pins	14	Nadeln	needles	13
Bonbons	sweets	14	Schlitten	sleds	12	Töpfe	pots	14
Spritzen	syringes	14	Dosen	cans	12	Engel	angels	11
Wale	whales	13	Palmen	palm trees	13	Sofas	sofas	14
Stempel	stamps	12	Würmer	worms	14	Blumen	flowers	11
Störche	storks	14	Pflaster	band aids	12	Tropfen	drops	12
Biber	beavers	13	Ampeln	traffic lights	13	Hemden	shirts	13
Bälle	balls	12	Katzen	cats	11	Wolken	clouds	11
Stiere	bulls	15	Waffeln	wafers	13	Besen	brooms	13
Kuchen	pies	10	Westen	vests	9	Mädchen	girls	8
Nüsse	nuts	13	Krücken	crutches	14	Pfeile	arrows	14
Füchse	foxes	13	Eimer	buckets	13	Boote	boats	12
Reifen	tires	11	Wölfe	wolves	12	Schaufeln	shovels	14
Zungen	tongues	14	Schüsseln	keys	15	Pandas	pandas	15
Bügel	coat hangers	15	Wannen	tubs	16	Reiben	rasps	16
Fenster	windows	9	Knochen	bones	11	Westen	vests	9
Brote	loaves of bread	14	Gänse	geese	14	Schnuller	pacifier	15
Zähne	teeth	11	Briefe	letters	11	Pilze	mushrooms	12
Räder	bikes	11	Handys	cell phones	11	Teufel	devils	11
Schnuller	pacifier	15	Zöpfe	braids	15	Fahnen	flags	12
Zirkel	compasses	14	Spinnen	spiders	13	Füße	feet	11
Bienen	bees	12	Hocker	stools	14	Gürtel	belts	12
Sofas	sofas	14	Hamster	hamsters	14	Stifte	pencils	15
Tropfen	drops	12	Noten	notes	11	Decken	blankets	12
Tafeln	boards	12	Bomben	bombs	12	Kühe	cows	12
Züge	trains	10	Schiffe	ships	11	Masken	masks	13
Duschen	showers	13	Robben	seals	11	Anker	anchors	13
Nester	nests	15	Kreisel	spinning tops	12	Fackeln	torches	14
Fackeln	torches	14	Tannen	christmas trees	14	Pinsel	brushes	13
Reiben	rasps	16	Siebe	strainers	17	Wiegen	cradles	16
Pandas	pandas	15	Wiegen	cradles	16	Ketten	chains	13
Kellen	scoops	17	Waagen	scales	17	Schwämme	sponges	16
Treppen	stairs	13	Brücken	bridges	11	Drachen	kites	13
Haken	hooks	12	Fahnen	flags	12	Birnen	pears	13
Fliegen	flies	12	Ohren	ears	10	Nägel	nails	13
Blusen	blouses	15	Kiwis	kiwis	15	Schnecken	snails	14
Brötchen	buns	12	Wurzeln	roots	10	Koffer	suitcases	11
Hirsche	stags	14	Eicheln	acorns	16	Kellen	scoops	17
Decken	blankets	12	Bürsten	brushes	15	Erbsen	peas	14
Hähne	roosters	16	Seile	ropes	15	Bügel	coat hangers	15
Becher	mugs	13	Stiefel	boots	13	Affen	monkeys	12
Netze	nets	12	Zelte	tents	12	Katzen	cats	11
Füße	feet	11	Kleider	dresses	12	Schwerter	swords	13
Nasen	noses	14	Scheren	scissors	15	Socken	socks	12
Erbsen	peas	14	Zangen	pliers	17	Raupen	caterpillars	15
Hände	hands	9	Fische	fish	11	Brötchen	buns	12
Kugeln	balls	12	Pizzen	pizzas	15	Fässer	barrels	14
Büsche	bushes	14	Deckel	lids	13	Hüte	hats	14
Affen	monkeys	12	Bänke	benches	13	Zwiebeln	onions	13
Zebras	zebras	14	Äpfel	apples	12	Kirschen	cherries	14
Zäune	fences	13	Enten	ducks	13	Äpfel	apples	12
Igel	hedgehogs	13	Monde	moons	13	Tannen	christmas trees	14
Zelte	tents	12	Mäuse	mice	13	Hirsche	stags	14
Fische	fish	11	Betten	beds	11	Schlitten	sleds	12
Socken	socks	12	Rehe	deer	14	Bonbons	sweets	14
Knochen	bones	11	Bären	bears	11	Hörner	horns	14
Bürsten	brushes	15	Möhren	carrots	14	Kegel	bowling pins	14
Kekse	cookies	14	Frösche	frogs	14	Scheren	scissors	15
Eimer	buckets	13	Schlangen	snakes	12	Uhren	clocks	12
Schuhe	shoes	10	Hunde	dogs	10	Pflaster	band aids	12
Torten	cakes	14	Hasen	bunnies	12	Sonnen	suns	15
Leitern	ladders	14	Birnen	pears	13	Zelte	tents	12
Schnecken	snails	14	Trauben	grapes	13	Hefte	books	14
Ziegen	goats	13	Schwäne	swans	14	Waagen	scales	17
Schirme	umbrellas	15	Sessel	chairs	13	Haie	sharks	13
Boote	boats	12	Mäntel	coats	15	Pizzen	pizzas	15
Eulen	owls	14	Schleifen	bows	14	Treppen	stairs	13
Teddys	teddy bears	16	Körbe	baskets	14	Hämmer	hammers	17
Geigen	violins	15	Ziegel	bricks	14	Zangen	pliers	17
Schiffe	ships	11	Häuser	houses	9	Tische	tables	12
Brücken	bridges	11	Engel	angels	11	Schleifen	bows	14
Tiger	tigers	11	Vögel	birds	11	Bären	bears	11
Uhren	clocks	12	Federn	feathers	12	Stühle	chairs	12
Jungen	boys	9	Bücher	books	9	Wurzeln	roots	10
Blumen	flowers	11	Mädchen	girls	8	Autos	cars	8
Münzen	coins	12	Schweine	pigs	12	Babys	babies	11
Gurken	cucumbers	14	Hefte	books	14	Vögel	birds	11
Messer	knives	10	Koffer	suitcases	11	Rosen	roses	12
Glocken	bells	12	Taschen	bags	12	Spritzen	syringes	14
Kirschen	cherries	14	Hühner	chicken	12	Fische	fish	11
Gürtel	belts	12	Lampen	lamps	12	Wale	whales	13
Löffel	spoons	13	Puppen	dolls	13	Füchse	foxes	13
Kissen	pillows	13	Trommeln	drums	13	Würmer	worms	14
Kiwis	kiwis	15	Tassen	cups	13	Büsche	bushes	14
Waffeln	wafers	13	Hämmer	hammers	17	Zöpfe	braids	15
Fässer	barrels	14	Gabeln	forks	16	Monde	moons	13
Sterne	stars	11	Pilze	mushrooms	12	Tassen	cups	13
Stifte	pencils	15	Pinsel	brushes	13	Mützen	caps	14
Hemden	shirts	13	Besen	brooms	13	Spiegel	mirrors	10
Bäume	trees	10	Autos	cars	8	Löwen	lions	10
Drachen	kites	13	Vasen	vases	15	Krücken	crutches	14
Scheren	scissors	15	Nadeln	needles	13	Sessel	chairs	13
Flaschen	bottles	11	Schlüssel	keys	11	Hasen	bunnies	12
Dosen	cans	12	Eier	eggs	11	Trommeln	drums	13
Sägen	saws	15	Schals	scarves	14	Pferde	horses	11
Kerzen	candles	11	Brillen	glasses	13	Jacken	jackets	13
Röcke	skirts	14	Angeln	fishing rods	14	Igel	hedgehogs	13
Gänse	geese	14	Hosen	pants	12	Kreisel	spinning tops	12
Blätter	leaves	11	Fäden	threads	12	Sterne	stars	12
Wolken	clouds	11	Babys	babies	11	Tüten	bags	13

Beine	legs	10	Teller	dishes	11	Netze	nets	12
Helme	helmets	14	Sonnen	suns	15	Blusen	blouses	15
Kreuze	crosses	13	Schafe	sheep	12	Leitern	ladders	14
Töpfe	pots	14	Schränke	cupboards	14	Zirkel	compasses	14
Ketten	chains	13	Bögen	bows	14	Helme	helmets	14
Katzen	cats	11	Jacken	jackets	13	Nasen	noses	14
Pizzen	pizzas	15	Tische	tables	12	Ziegel	bricks	14
Schaufeln	shovels	14	Pfannen	pans	15	Kiwis	kiwis	15
Pflaumen	plums	15	Nägel	nails	13	Würste	sausages	14
Gläser	glasses	12	Rosen	roses	12	Bögen	bows	14
Tannen	christmas trees	14	Schwerter	swords	13	Möhren	carrots	14
Ringe	rings	12	Kronen	crowns	11	Zungen	tongues	14
Ampeln	traffic lights	13	Käse	loaves of cheese	11	Hamster	hamsters	14
Löwen	lions	10	Herzen	hearts	9	Züge	trains	10
Würfel	dice	13	Pfeile	arrows	14	Schirme	umbrellas	15
Stühle	chairs	12	Esel	donkeys	12	Blätter	leaves	11
Palmen	palm trees	13	Anker	anchors	13	Handys	cell phones	11
Flöten	flutes	15	Kämme	combs	17	Siebe	strainers	17
Muscheln	clams	13	Pfeifen	pipes	14	Schwäne	swans	14
Würmer	worms	14	Hörner	horns	14	Lippen	lips	12
Knöpfe	buttons	14	Raupen	caterpillars	15	Eicheln	acorns	16
Krücken	crutches	14	Bonbons	sweets	14	Eier	eggs	11
Zwiebeln	onions	13	Spritzen	syringes	14	Rehe	deer	14
Kühe	cows	12	Wale	whales	13	Sägen	saws	15
Pflaster	band aids	12	Stempel	stamps	12	Käse	loaves of cheese	11
Teufel	devils	11	Störche	storks	14	Zäune	fences	13
Kegel	bowling pins	14	Biber	beavers	13	Stiefel	boots	13
Schlitten	sleds	12	Bälle	balls	12	Noten	notes	11
Kannen	jugs	16	Stiere	bulls	15	Wannen	tubs	16
Ruder	paddles	12	Kuchen	pies	10	Schiffe	ships	11
Lippen	lips	12	Nüsse	nuts	13	Bänke	benches	13
Hüte	hats	14	Füchse	foxes	13	Geigen	violins	15
Fahnen	flags	12	Reifen	tires	11	Störche	storks	14
Hamster	hamsters	14	Zungen	tongues	14	Körbe	baskets	14
Schüsseln	keys	15	Bügel	coat hangers	15	Dosen	cans	12
Augen	eyes	8	Fenster	windows	9	Jungen	boys	9
Hocker	stools	14	Brote	loaves of bread	14	Trauben	grapes	13
Masken	masks	13	Zähne	teeth	11	Frösche	frogs	14
Türen	doors	10	Räder	bikes	11	Esel	donkeys	12
Wannen	tubs	16	Schnuller	pacifier	15	Kämme	combs	17
Würste	sausages	14	Zirkel	compasses	14	Muscheln	clams	13
Wölfe	wolves	12	Bienen	bees	12	Haken	hooks	12
Spinnen	spiders	13	Sofas	sofas	14	Knöpfe	buttons	14
Wurzeln	roots	10	Tropfen	drops	12	Glocken	bells	12
Briefe	letters	11	Tafeln	boards	12	Kerzen	candles	11
Pferde	horses	11	Züge	trains	10	Hände	hands	9
Bomben	bombs	12	Duschen	showers	13	Teller	dishes	11
Mützen	caps	14	Nester	nests	15	Stempel	stamps	12
Zöpfe	braids	15	Fackeln	torches	14	Ampeln	traffic lights	13
Waagen	scales	17	Reiben	rasps	16	Eulen	owls	14
Siebe	strainers	17	Pandas	pandas	15	Vasen	vases	15
Zangen	pliers	17	Kellen	scoops	17	Pflaumen	plums	15
Seile	ropes	15	Treppen	stairs	13	Robben	seals	11
Deckel	lids	13	Haken	hooks	12	Bürsten	brushes	15
Handys	cell phones	11	Fliegen	flies	12	Bäume	trees	10
Schwämme	sponges	16	Blusen	blouses	15	Seile	ropes	15
Stiefel	boots	13	Brötchen	buns	12	Angeln	fishing rods	14
Kleider	dresses	12	Hirsche	stags	14	Briefe	letters	11
Westen	vests	9	Decken	blankets	12	Ohren	ears	10
Eicheln	acorns	16	Hähne	roosters	16	Kekse	cookies	14
Kreisel	spinning tops	12	Becher	mugs	13	Schränke	cupboards	14
Ohren	ears	10	Netze	nets	12	Häuser	houses	9
Noten	notes	11	Füße	feet	11	Bücher	books	9
Tüten	bags	13	Nasen	noses	14	Hähne	roosters	16
Wiegen	cradles	16	Erbsen	peas	14	Schals	scarves	14
Spiegel	mirrors	10	Hände	hands	9	Betten	beds	11
Robben	seals	11	Kugeln	balls	12	Fenster	windows	9
Haie	sharks	13	Büsche	bushes	14	Gabeln	forks	16

Table 5: List of word/pictures combinations used for the filler trials

Critical Word	translation	Shown Object 1	translation	Shown Object 2	translation	Shown Object 3	translation
Trichter	funnels	Trichter	funnels	blank		blank	
Ballons	balloons	Ballons	balloons	blank		blank	
Trompeten	trumpets	Trompeten	trumpets	blank		blank	
Tastaturen	keyboards	Tastaturen	keyboards	blank		blank	
Ameisen	ants	Steine	rocks	blank		blank	
Steine	rocks	Locher	hole punches	blank		blank	
Locher	hole punches	Pullover	pullover	blank		blank	
Pullover	pullover	Ameisen	ants	blank		blank	
Drucker	printers	Drucker	printers	Trichter	funnels	Regale	shelves
Mikrofone	microphones	Mikrofone	microphones	Ballons	balloons	Pakete	parcels
Strohhalme	straws	Strohhalme	straws	Trompeten	trumpets	Wippen	seesaws
Klaviere	pianos	Klaviere	pianos	Tastaturen	keyboards	Traktoren	tractors
Vorhänge	curtains	Vorhänge	curtains	Ameisen	ants	Schmetterlinge	butterflies
Rucksäcke	backpacks	Rucksäcke	backpacks	Steine	rocks	Hecken	hedges
Laternen	lanterns	Laternen	lanterns	Locher	hole punches	Medaillen	medals
Orangen	oranges	Orangen	oranges	Pullover	pullover	Mikroskope	microscopes
Helikopter	helicopters	Helikopter	helicopters	blank		blank	
Elefanten	elephants	Elefanten	elephants	blank		blank	
Pinguine	penguins	Pinguine	penguins	blank		blank	
Raketen	rockets	Raketen	rockets	blank		blank	
Krawatten	ties	Tomaten	tomatoes	blank		blank	
Tomaten	tomatoes	Krokodile	crocodiles	blank		blank	
Krokodile	crocodiles	Bananen	bananas	blank		blank	
Bananen	bananas	Krawatten	ties	blank		blank	
Delphine	dolphins	Delphine	dolphins	Drucker	printers	Brunnen	wells
Regale	shelves	Regale	shelves	Mikrofone	microphones	Käfige	cages
Pakete	parcels	Pakete	parcels	Strohhalme	straws	Schubladen	drawers
Wippen	seesaws	Wippen	seesaws	Klaviere	pianos	Telefone	telephones
Traktoren	tractors	Traktoren	tractors	Vorhänge	curtains	Kartoffeln	chestnuts
Schmetterlinge	butterflies	Schmetterlinge	butterflies	Rucksäcke	backpacks	Harfen	harps
Hecken	hedges	Hecken	hedges	Laternen	lanterns	Streichhölzer	matches
Medaillen	medals	Medaillen	medals	Orangen	oranges	Kastanien	pianos
Mikroskope	microscopes	Mikroskope	microscopes	Helikopter	helicopters	blank	
Brunnen	wells	Brunnen	wells	Elefanten	elephants	blank	
Käfige	cages	Käfige	cages	Pinguine	penguins	blank	
Schubladen	drawers	Schubladen	drawers	Raketen	rockets	blank	
Telefone	telephones	Kartoffeln	potatoes	Krawatten	ties	blank	
Kartoffeln	potatoes	Harfen	harps	Tomaten	tomatoes	blank	
Harfen	harps	Streichhölzer	matches	Krokodile	crocodiles	blank	

Streichhölzer	matches	Telefone	telephones	Bananen	bananas	blank	
Kastanien	chestnuts	Kastanien	chestnuts	Delphine	dolphins	blank	
Raben	ravens	Raben	ravens	Regale	shelves	blank	
Aktenordner	folders	Aktenordner	folders	Pakete	parcels	blank	
Giraffen	giraffes	Giraffen	giraffes	Wippen	seesaws	blank	
Pfirsiche	peaches	Pfirsiche	peaches	Traktoren	tractors	blank	
Herde	cookers	Herde	cookers	Schmetterlinge	butterflies	blank	
Schinken	hams	Schinken	hams	Hecken	hedges	blank	
Wimpel	pennants	Wimpel	pennants	Medaillen	medals	blank	
Kameras	cameras	Kameras	cameras	Mikroskope	microscopes	blank	
Schaukeln	swings	Schaukeln	swings	Brunnen	wells	blank	
Käfer	beetles	Käfer	beetles	Käfige	cages	blank	
Schildkröten	turtles	Schildkröten	turtles	Schubladen	drawers	blank	
Tacker	staplers	Kräne	cranes	Telefone	telephones	blank	
Lupen	magnifiers	Tacker	staplers	Kartoffeln	potatoes	blank	
Lineale	rulers	Lupen	magnifiers	Harfen	harps	blank	
Hufeisen	horseshoes	Lineale	rulers	Streichhölzer	matches	blank	
Schlösser	castles	Hufeisen	horseshoes	Kastanien	chestnuts	blank	
Kräne	cranes	Schlösser	castles	Raben	ravens	blank	
Melonen	melons	Melonen	melons	blank		blank	
Matratzen	mattresses	Matratzen	mattresses	blank		blank	
Magnete	magnets	Magnete	magnets	blank		blank	
Fotos	photos	Fotos	photos	blank		blank	
Laptops	laptops	Laptops	laptops	blank		blank	
Kakteen	cacti	Kakteen	cacti	blank		blank	
Handtücher	towels	Handtücher	towels	blank		blank	
Kutschen	carriages	Kutschen	carriages	blank		blank	
Gitarren	guitars	Särge	coffins	blank		blank	
Rechen	rakes	Gitarren	guitars	blank		blank	
Rasenmäher	lawn mowers	Rechen	rakes	blank		blank	
Särge	coffins	Rasenmäher	lawn mowers	blank		blank	
Gespenster	ghosts	Gespenster	ghosts	Aktenordner	folders	Raben	ravens
Flugzeuge	planes	Flugzeuge	planes	Giraffen	giraffes	Aktenordner	folder
Schlafsäcke	sleeping bags	Schlafsäcke	sleeping bags	Pfirsiche	peaches	Giraffen	giraffes
Computer	computers	Computer	computers	Herde	cookers	Pfirsiche	peaches
Burgen	castles	Burgen	castles	Schinken	hams	Herde	cookers
Waschmaschinen	washing machines	Waschmaschinen	washing machines	Wimpel	pennants	Schinken	hams
Clowns	clowns	Clowns	clowns	Kameras	cameras	Wimpel	pennants
Zigaretten	cigarettes	Zigaretten	cigarettes	Schaukeln	swings	Kameras	cameras
Stecker	plugs	Stecker	plugs	Käfer	beetles	Schaukeln	swings
Murmeln	marbles	Murmeln	marbles	Schildkröten	turtles	Käfer	beetles
Eichhörnchen	squirrels	Eichhörnchen	squirrels	Tacker	staplers	blank	
Zitronen	lemons	Zitronen	lemons	Lupen	magnifiers	blank	
Papageien	parrots	Papageien	parrots	Lineale	rulers	blank	
Narzissen	daffodils	Narzissen	daffodils	Hufeisen	horseshoes	blank	
Kamine	fireplaces	Kamine	fireplaces	Schlösser	castles	blank	
Eidechsen	lizards	Eidechsen	lizards	Kräne	cranes	blank	
Statuen	statues	Statuen	statues	Melonen	melons	blank	
Skulpturen	sculptures	Skulpturen	sculptures	Matratzen	mattresses	blank	
Schneemänner	snowmen	Steine	rocks	Magnete	magnets	blank	
Teppiche	rugs	Schneemänner	snowmen	Fotos	photos	blank	
Trichter	funnels	Teppiche	rugs	Laptops	laptops	blank	
Ballons	balloons	Trichter	funnels	Kakteen	cacti	blank	
Trompeten	trumpets	Ballons	balloons	Handtücher	towels	blank	
Tastaturen	keyboards	Trompeten	trumpets	Kutschen	carriages	blank	
Ameisen	ants	Tastaturen	keyboards	Gitarren	guitars	blank	
Steine	rocks	Ameisen	ants	Rechen	rakes	blank	
Locher	hole punches	Locher	hole punches	blank		blank	
Pullover	pullover	Pullover	pullover	blank		blank	
Drucker	printers	Drucker	printers	blank		blank	
Mikrofone	microphones	Mikrofone	microphones	blank		blank	
Strohhalme	straws	Strohhalme	straws	blank		blank	
Klaviere	pianos	Klaviere	pianos	blank		blank	
Vorhänge	curtains	Tomaten	tomatoes	blank		blank	
Rucksäcke	backpacks	Vorhänge	curtains	blank		blank	
Laternen	lanterns	Rucksäcke	backpacks	blank		blank	
Orangen	oranges	Laternen	lanterns	blank		blank	
Helikopter	helicopters	Orangen	oranges	blank		blank	
Elefanten	elephants	Helikopter	helicopters	blank		blank	
Pinguine	penguins	Elefanten	elephants	blank		blank	
Raketen	rockets	Pinguine	penguins	blank		blank	
Krawatten	ties	Raketen	rockets	blank		blank	
Tomaten	tomatoes	Krawatten	ties	blank		blank	
Krokodile	crocodiles	Krokodile	crocodiles	Rasenmäher	lawn mowers	Schildkröten	turtles
Bananen	bananas	Bananen	bananas	Särge	coffins	Tacker	staplers
Delphine	dolphins	Delphine	dolphins	Gespenster	ghosts	Lupen	rulers
Regale	shelves	Regale	shelves	Flugzeuge	planes	Lineale	lanterns
Pakete	parcels	Pakete	parcels	Schlafsäcke	sleeping bags	Hufeisen	horseshoes
Wippen	seesaws	Wippen	seesaws	Computer	computers	Schlösser	castles
Traktoren	tractors	Traktoren	tractors	Burgen	castles	Kräne	cranes
Schmetterlinge	butterflies	Schmetterlinge	butterflies	Waschmaschinen	washing machines	Melonen	melons
Hecken	hedges	Hecken	hedges	blank		blank	
Medaillen	medals	Medaillen	medals	blank		blank	
Mikroskope	microscopes	Mikroskope	microscopes	Zigaretten	cigarettes	blank	
Brunnen	wells	Brunnen	wells	Stecker	plugs	blank	
Käfige	cages	Käfige	cages	Murmeln	marbles	Matratzen	mattresses
Schubladen	drawers	Schubladen	drawers	Eichhörnchen	printers	Magnete	magnets
Harfen	harps	Magnete	magnets	blank		blank	
Magnete	magnets	Fotos	photos	blank		blank	
Fotos	photos	Streichhölzer	matches	Zitronen	lemons	blank	
Streichhölzer	matches	Harfen	harps	Papageien	parrots	blank	
Kastanien	chestnuts	Kastanien	chestnuts	blank		blank	
Raben	ravens	Raben	ravens	blank		blank	
Aktenordner	folders	Herde	cookers	blank		blank	
Giraffen	giraffes	Aktenordner	folders	blank		blank	
Pfirsiche	peaches	Narzissen	daffodils	Kamine	fireplaces	blank	
Herde	cookers	Pfirsiche	peaches	Eidechsen	printers	blank	
Schinken	hams	Schinken	hams	Statuen	statues	blank	
Wimpel	pennants	Wimpel	pennants	Skulpturen	sculptures	blank	
Kameras	cameras	Kameras	cameras	Schneemänner	snowmen	Fotos	photos
Schaukeln	swings	Schaukeln	swings	Teppiche	rugs	Laptops	laptops
Käfer	beetles	Käfer	beetles	blank		blank	
Schildkröten	turtles	Schildkröten	turtles	blank		blank	
Tacker	staplers	Tacker	staplers	Clowns	clowns	blank	
Lupen	magnifiers	Lupen	magnifiers	Trompeten	trumpets	blank	
Lineale	rulers	Lineale	rulers	Tastaturen	keyboards	Kakteen	cacti
Hufeisen	horseshoes	Hufeisen	horseshoes	Ameisen	ants	Handtücher	towels
Schlösser	castles	Kräne	cranes	blank		blank	
Kräne	cranes	Melonen	melons	blank		blank	
Melonen	melons	Matratzen	mattresses	Steine	rocks	blank	
Matratzen	mattresses	Schlösser	castles	Locher	hole punches	blank	
Telefone	telephones	Telefone	telephones	blank		blank	
Kartoffeln	potatoes	Kartoffeln	potatoes	blank		blank	
Laptops	laptops	Kutschen	carriages	blank		blank	
Kakteen	cacti	Laptops	laptops	blank		blank	

Handtücher	towels	Kakteen	cacti	Pullover	pullover	blank	
Kutschen	carriages	Handtücher	towels	Drucker	printers	blank	
Gitarren	guitars	Gitarren	guitars	Mikrofone	microphones	blank	
Rechen	rakes	Rechen	rakes	Strohhalme	straws	blank	
Rasenmäher	lawn mowers	Rasenmäher	lawn mowers	Klaviere	pianos	Kutschen	carriages
Särge	coffins	Särge	coffins	Vorhänge	curtains	Gitarren	guitars
Gespenster	ghosts	Gespenster	ghosts	blank		blank	
Flugzeuge	planes	Flugzeuge	planes	blank		blank	
Schlafsäcke	sleeping bags	Schlafsäcke	sleeping bags	Rucksäcke	backpacks	blank	
Computer	computers	Computer	computers	Laternen	lanterns	blank	
Burgen	castles	Burgen	castles	Orangen	oranges	Rechen	rakes
Waschmaschinen	washing machines	Waschmaschinen	washing machines	Helikopter	helicopters	Rasenmäher	lawn mowers
Clowns	clowns	Murmeln	marbles	blank		blank	
Zigaretten	cigarettes	Clowns	clowns	blank		blank	
Stecker	plugs	Zigaretten	cigarettes	Elefanten	elephants	blank	
Murmeln	marbles	Stecker	plugs	Pinguine	penguins	blank	
Eichhörnchen	squirrels	Eichhörnchen	squirrels	blank		blank	
Zitronen	lemons	Zitronen	lemons	blank		blank	
Papageien	parrots	Eidechsen	lizards	blank		blank	
Narzissen	daffodils	Papageien	parrots	blank		blank	
Kamine	fireplaces	Narzissen	daffodils	Raketen	rockets	blank	
Eidechsen	lizards	Kamine	fireplaces	Krawatten	ties	blank	
Statuen	statues	Statuen	statues	Tomaten	tomatoes	blank	
Skulpturen	sculptures	Skulpturen	sculptures	Krokodile	crocodiles	blank	
Schneemänner	snowmen	Schneemänner	snowmen	Bananen	bananas	Särge	coffins
Teppiche	rugs	Teppiche	rugs	Delphine	dolphins	Gespenster	ghosts

Table 6: Experiment 1: The mean accuracy (with standard deviation) per filler category (known vs. unknown truth-value) and per group. For the sake of ease of exposition, the logical responses are throughout counted as "accurate" for *some* and for bare numerals (based on the *at least* reading). For the quantifier *some* the filler trials are further divided into those whose truth-value is known and unambiguous (i.e. not dependent on the reading), unknown and unambiguous, known but ambiguous (i.e. cases where the speaker has a full access but the stronger alternative is true; here "inaccurate" response indicates a weak pragmatic interpretation), unknown and ambiguous (the speaker has a partial access and the stronger alternative is true in the visible cards; here "inaccurate" response indicates a strong pragmatic interpretation).

Quantifier	*filler truth-value*	*Logicians* (N=33)	*W Pragmatists* (N=10)	*St Prag* (N=1)	*Other* (N=3)
Some	*Known (unambiguous)*	97.52 (2.74)	96.67 (1.17)	96.30	96.25 (0.08)
	Unknown (unambiguous)	100.00	97.50 (7.91)	100.00	58.33 (14.3)
	Unknown (ambiguous)	98.48 (6.06)	97.50 (7.91)	0.00	100.00
	Known (ambiguous)	95.80 (17.14)	8.00 (13.98)	0.00	50.00 (50.00)
All	*Known*	97.05 (4.35)	97.30 (3.37)	100.00	98.2 (1.56)
	Unknown	85.86 (23.61)	96.67 (10.54)	100.00	55.56 (50.92)
No	*Known*	96.97 (3.25)	97.94 (3.68)	100.00	98.99 (1.75)
	Unknown	69.70 (25.84)	71.67 (20.86)	83.33	72.22 (19.25)
More than	*Known*	98.40 (3.04)	98.20 (2.90)	100.00	96.08 (3.40)
	Unknown	94.95 (12.14)	96.67 (10.54)	66.67	33.33
Fewer than	*Known*	83.49 (14.81)	80.63 (19.20)	87.50	91.37 (9.46)
	Unknown	61.62 (26.26)	50.83 (33.21)	33.33	47.22 (41.11)
Bare numerals	*Known*	82.65 (6.73)	84.85 (5.34)	83.33	83.33
	Unknown	99.24 (4.35)	93.33 (21.08)	100.00	8.33 (14.43)

1 Instructions, Feedback, and Exercises - in German

1.1 Main Instruction - Screen 1

In diesem Experiment testen wir dein Verständnis von gesprochenen Sätzen und deine Aufmerksamkeit beim Betrachten von Bildern.

In jedem Durchgang siehst du eine Spielsituation, bei der du und ein weiterer Spieler oder eine weitere Spielerin Karten auf dem Tisch und außerhalb des Tisches sehen können.

Alle sichtbaren Karten, sowohl die auf dem Tisch als auch außerhalb von diesem, befinden sich im Spiel. Die Karten auf dem Tisch sind immer offen, die Karten außerhalb des Tisches können verdeckt oder offen sein. Bilder auf verdeckten Karten sind euch beiden nicht bekannt.

Auf einer Karte befinden sich maximal zwei Objekte. Die Karten in einer Runde zeigen bis zu drei verschiedene Typen von Objekten. Bitte beachte, dass es auch leere Karten geben kann.

Jede Spielrunde wird von einem gesprochenen Satz deines Mitspielers oder deiner Mitspielerin begleitet, welchen er oder sie in einem vorherigen Versuch aufgenommen hat. Dabei gibt es Aussagen, welche die Spielsituation angemessen beschreiben, und welche, die unangemessen sind. Bei manchen der unangemessen Sätze macht er oder sie einen Fehler, manchmal ist es aber auch so, dass er oder sie bewusst einen unangemessenen Satz äußert.

Im Anschluss an eine Spielrunde erscheinen die Wörter 'JA' und 'NEIN' jeweils rechts und links, allerdings auf wechselnden Seiten. Sie zeigen dir an, auf welche Taste du drücken sollst, um zu antworten.

Deine Aufgabe ist es zu bewerten, welche der Aussagen angemessen und welche unangemessen sind.

Wenn du keine Fragen hast, kannst du mit einem Tastendruck fortfahren.

1.1.1 Main Instruction - Screen 2

Du spielst deine Runden mit Lena.

1.1.2 Main Instruction - Screen 3

Zunächst gibt es eine kurze Übung, um dich an die Aufgabe zu gewöhnen. Drücke bitte eine Taste, sobald du bereit bist.

1.2 Response Feedback for Exercises

1.2.1 Feedback Response For Correct Responses

Du hast richtig geantwortet.

1.2.2 Feedback Response For False Responses

Du hast nicht richtig geantwortet.

1.2.3 Feedback Response For Missed Responses

Bitte beantworte die nächste Frage!

1.3 Exercises in German

1.3.1 Exercise 1

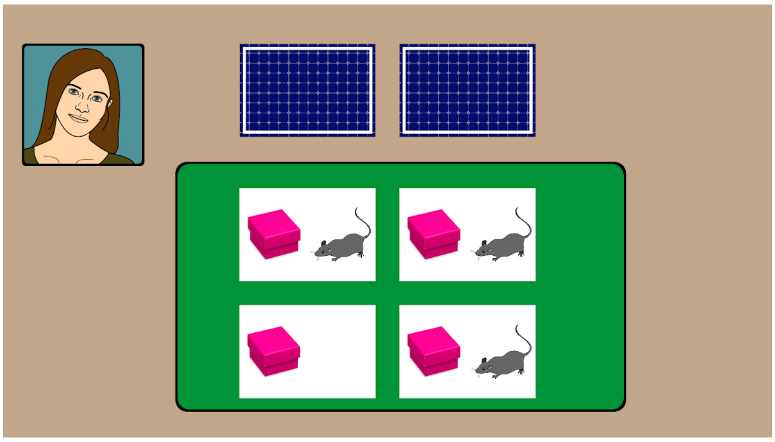

Lena: “Alle Karten auf dem Tisch enthalten Kästchen.”

Du hast richtig geantwortet. / Du hast nicht richtig geantwortet. / Bitte beantworte die nächste Frage!

Alle Karten auf dem Tisch, die von Lena gesehen werden können, enthalten Kästchen. Es ist für Lena also angemessen, diesen Satz zu äußern. Wenn dir das klar ist, kannst du mit einem Tastendruck fortfahren.

1.3.2 Exercise 2

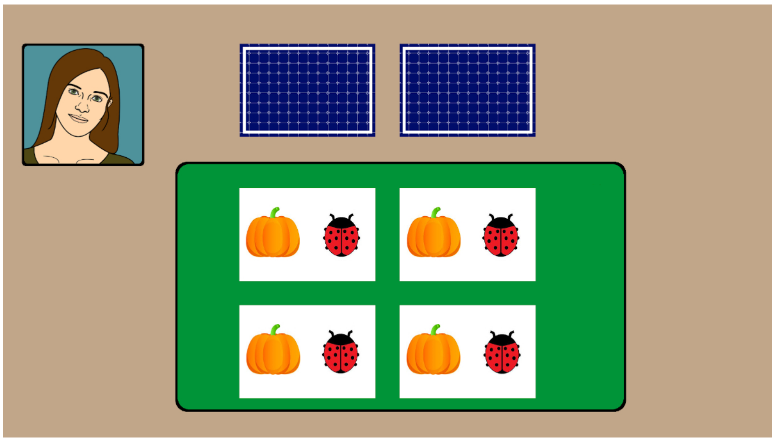

Lena: “Alle Karten im Spiel enthalten Marienkäfer.”

Du hast richtig geantwortet. / Du hast nicht richtig geantwortet. / Bitte beantworte die nächste Frage!

Lena kann nicht wissen, ob alle Karten im Spiel Marienkäfer enthalten, weil die zwei zusätzlichen Karten neben dem Tisch verdeckt sind. Damit ist es für Lena nicht angemessen, diesen Satz zu äußern, denn eine Aussage kann nur dann angemessen sein, wenn man weiß, dass diese wahr ist. Wenn dir das klar ist, kannst du mit einem Tastendruck fortfahren.

1.3.3 Exercise 3

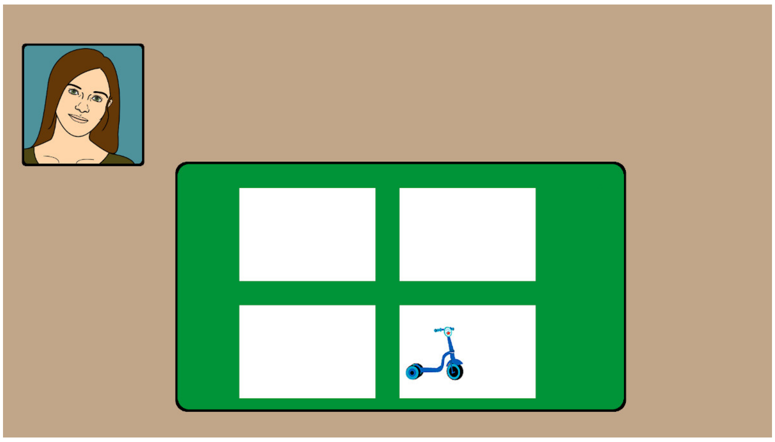

Lena: “Keine Karten im Spiel enthalten Roller.”

Du hast richtig geantwortet. / Du hast nicht richtig geantwortet. / Bitte beantworte die nächste Frage!

Es ist nicht wahr, dass keine Karte im Spiel Roller enthält. Lena kann sehen, dass auf mindestens einer Karte ein Roller ist. Damit ist es für Lena nicht angemessen, diesen Satz zu äußern, weil der Satz falsch ist. Wenn dir das klar ist, kannst du mit einem Tastendruck fortfahren.

1.3.4 Exercise 4

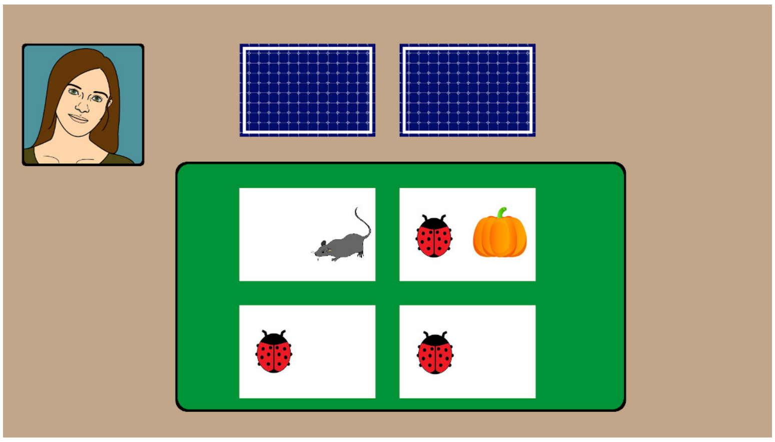

Lena: “Weniger als zwei Karten im Spiel enthalten Ratten.”

Du hast richtig geantwortet. / Du hast nicht richtig geantwortet. / Bitte beantworte die nächste Frage!

Lena kann sehen, dass mindestens eine Karte im Spiel eine Ratte enthält, aber sie kann nicht sehen, ob es auf den verdeckten Karten mehr Ratten gibt. Also kann sie nicht wissen, ob weniger als zwei Karten im Spiel Ratten enthalten. Damit ist es für Lena nicht angemessen, diesen Satz zu äußern.

Die nächsten Übungen werden keine Erklärungen mehr enthalten. Tauchen während der Übung Unklarheiten auf, kannst du vor dem Hauptexperiment die Versuchsleitung fragen. Wenn dir das klar ist, kannst du mit einem Tastendruck fortfahren.

1.3.5 Exercise 5

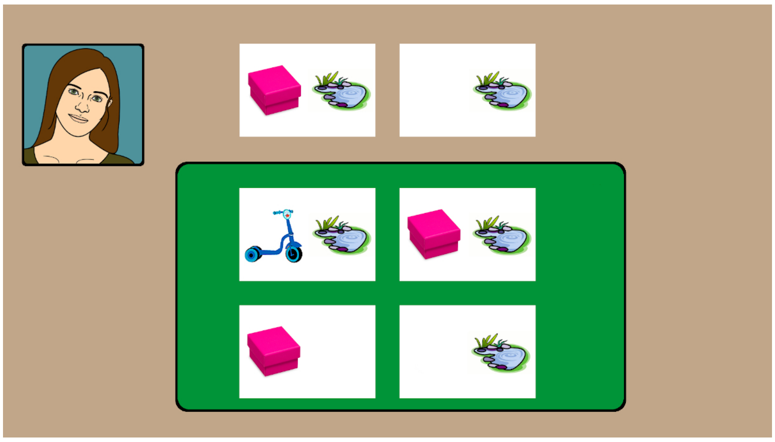

Lena: “Mehr als zwei Karten auf dem Tisch enthalten Teiche .”

Du hast richtig geantwortet. / Du hast nicht richtig geantwortet. / Bitte beantworte die nächste Frage!

1.3.6 Exercise 6

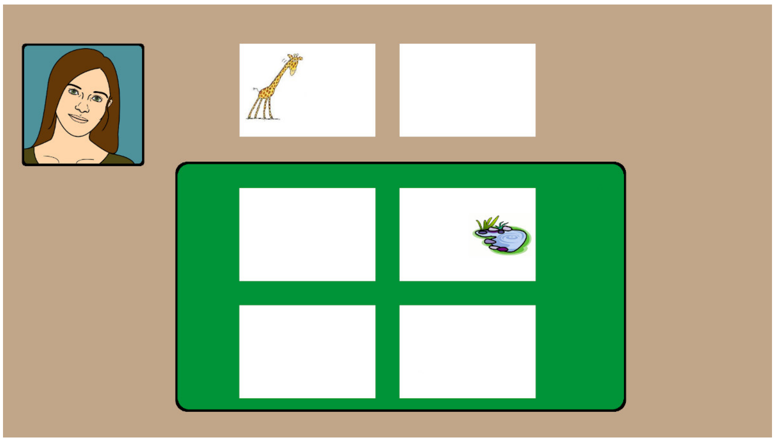

Lena: “Keine Karten auf dem Tisch enthalten Kürbisse .”

Du hast richtig geantwortet. / Du hast nicht richtig geantwortet. / Bitte beantworte die nächste Frage!

1.3.7 Exercise 7

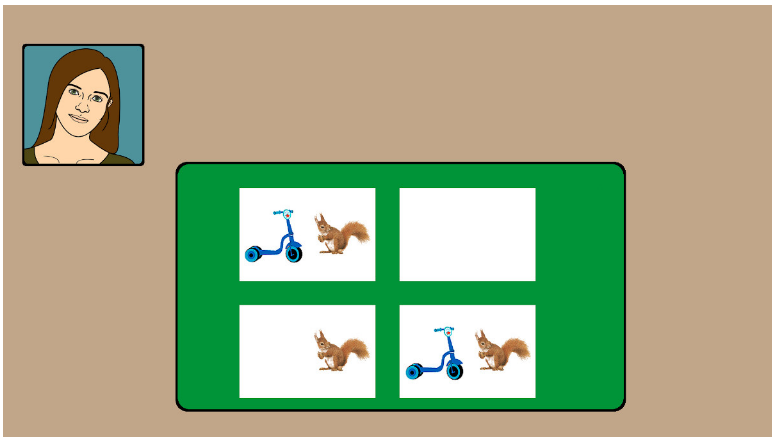

Lena: “Mehr als drei Karten im Spiel enthalten Teiche.”

Du hast richtig geantwortet. / Du hast nicht richtig geantwortet. / Bitte beantworte die nächste Frage!

1.3.8 Exercise 8

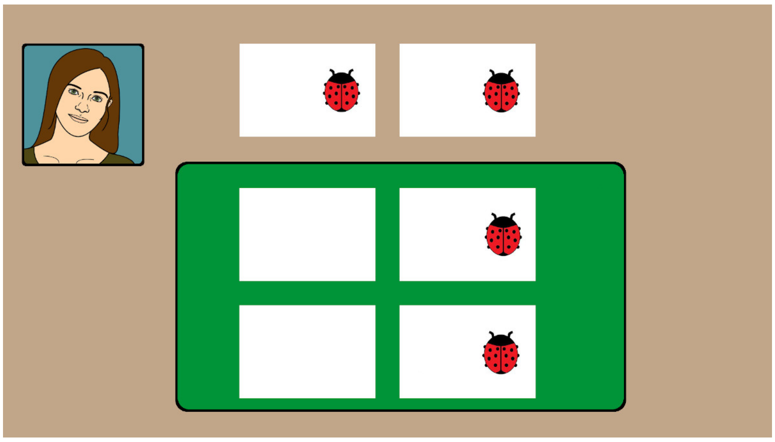

Lena: “Weniger als drei Karten auf dem Tisch enthalten Marienkäfer.”

Du hast richtig geantwortet. / Du hast nicht richtig geantwortet. / Bitte beantworte die nächste Frage!

1.4 End of Exercises

Das war die Übung. Wenn du keine weiteren Fragen hast, geht das Hauptexperiment mit einem Tastendruck los. Bitte beachte, dass du während des Experimentes kein Feedback bekommen wirst.

Dieser Teil ist in sieben Blöcke unterteilt, zwischen denen du jeweils eine kurze Pause einlegen kannst.

2 Translated Instructions

2.1 Main Instruction - Screen 1

In this experiment we text your understanding of spoken sentences and your attention while observing pictures.

In every round, you will see a game scenario, within which you and another player will be able to see cards on a table and outside of the table.

All cards seen, the ones on the table as well the ones outside of it, are part of the game. The cards on the table are always dealt with their face side up, the cards outside the table can be dealt with their face side up or with their backside up. The pictures on the cards with their backside up are unknown to both of you.

Every card shows a maximum of two objects. The cards in every round show up to three different types of pictures. Please keep in mind, that it is possible for cards to be blank.

Every round is accompanied by a spoken sentence of your co-player that was recorded during a previous experiment. There are statements that are describing the situation appropriately and statements that are inappropriate. In the case of inappropriate statements your co-player might have made a mistake, but sometimes he or she deliberately chose an inappropriate statement.

After every round, you will see the words 'YES' and 'NO' on the right and left side and their location will be assigned randomly after each round. They indicate the button you need to press to give your answer.

Now press the corresponding button to answer the question if your co-player described the scenario in an appropriate manner.

If you have no further questions, you may proceed by pressing a button.

2.1.1 Main Instruction - Screen 2

You are playing your rounds with Lena.

2.1.2 Main Instruction - Screen 3

First, there will be a short exercise to get you used to the task. Please press a button if you are ready.

2.2 Response Feedback for Exercises

2.2.1 Feedback Response For Correct Responses

Your answer is correct.

2.2.2 Feedback Response For False Responses

Your answer is not correct.

2.2.3 Feedback Response For Missed Responses

Please give an answer to the next question!

2.3 Exercises

2.3.1 Exercise 1

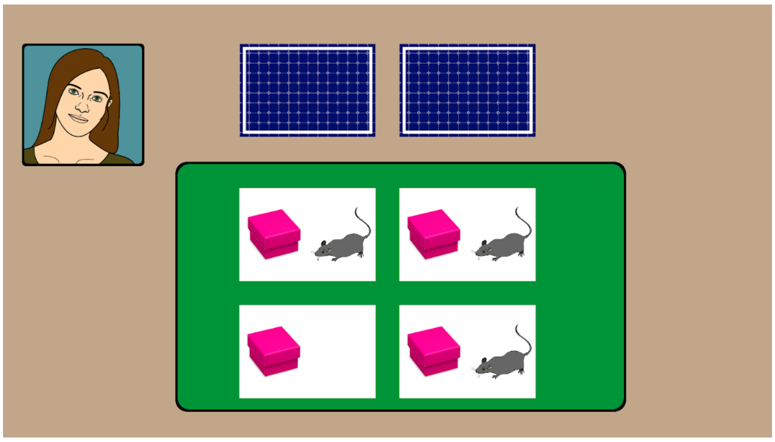

Lena: “All cards on the table contain boxes.”

Your answer is correct. / Your answer is not correct. / Please give an answer to the next question!

All cards on the table can be seen by Lena and contain boxes. Thus it is appropriate for Lena to utter this sentence. If you understood this, you may proceed by pressing a button.

2.3.2 Exercise 2

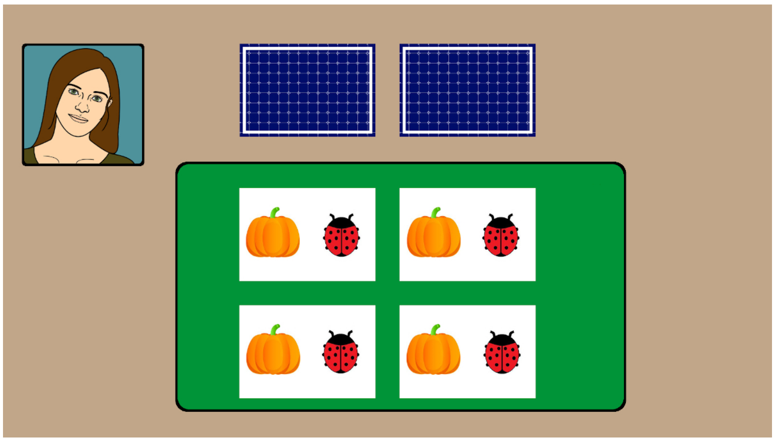

Lena: “All cards in the game contain ladybugs.”

Your answer is correct. / Your answer is not correct. / Please give an answer to the next question!

Lena cannot know whether all cards in the game contain ladybugs or not because the two additional cards next to the table are turned over. Thus, it is not appropriate for Lena to utter this sentence, since a statement can only be appropriate if one knows that it is true. If you understood this, you may proceed by pressing a button.

2.3.3 Exercise 3

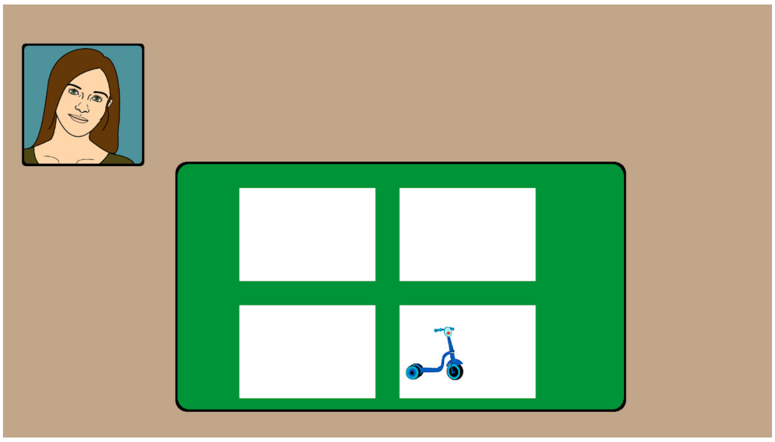

Lena: "No cards in the game contain scooters."

Your answer is correct. / Your answer is not correct. / Please give an answer to the next question!

It is not true that no card in the game contains a scooter. Lena can see that at least one card contains a scooter. Thus, it is not appropriate for Lena to utter this sentence because the sentence is false. If you understood this, you may proceed by pressing a button.

2.3.4 Exercise 4

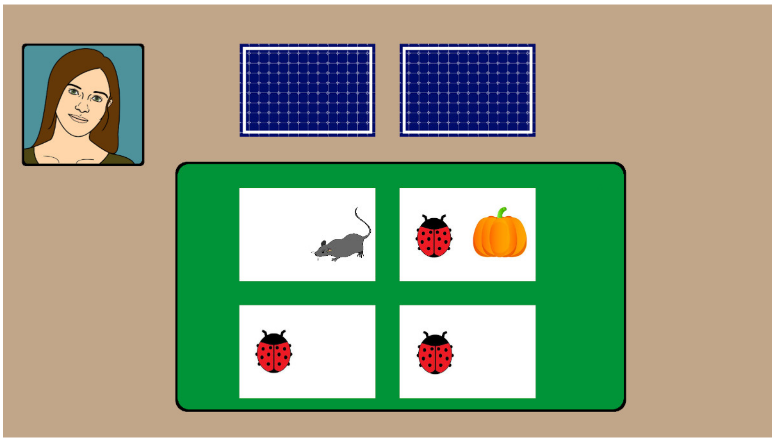

Lena: "Less than two cards in the game contain rats."

Your answer is correct. / Your answer is not correct. / Please give an answer to the next question!

Lena can see that at least one card in the game contains a rat but she cannot see if there are more rats on the cards that are face down. So, she cannot know whether less than two cards in the game contain rats or not. Thus, it is not appropriate for Lena to utter this sentence. If you understood this, you may proceed by pressing a button.

The next exercises will not have explanations anymore. If questions arise during the exercises, you can ask experimenters to clarify them before the start of the main experiments. If you understood this, you may proceed by pressing a button.

2.3.5 Exercise 5

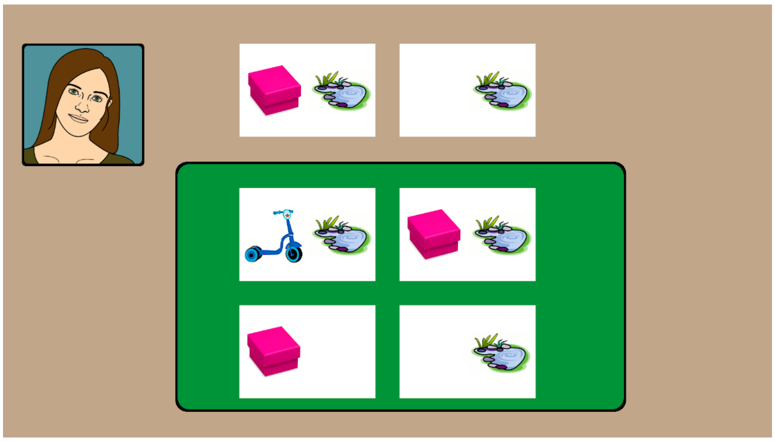

Lena: “More than two cards on the table contain ponds.”

Your answer is correct. / Your answer is not correct. / Please give an answer to the next question!

2.3.6 Exercise 6

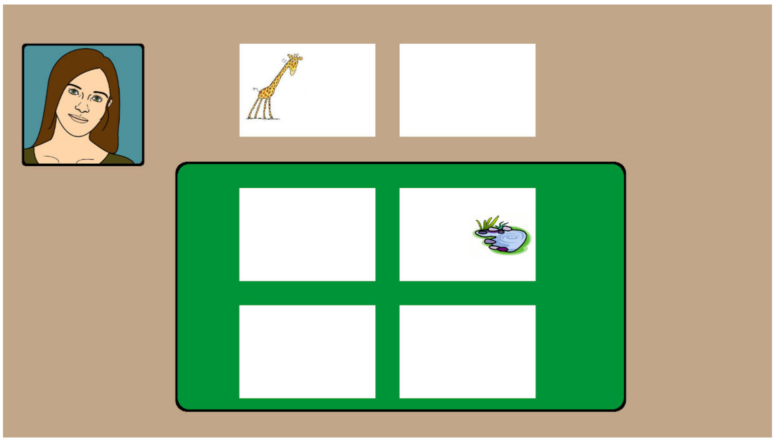

Lena: “No cards on the table contain pumpkins.”

Your answer is correct. / Your answer is not correct. / Please give an answer to the next question!

2.3.7 Exercise 7

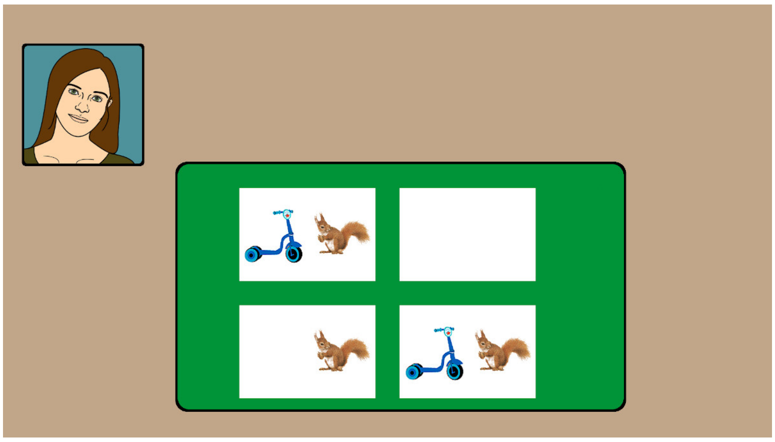

Lena: “More than three cards in the game contain ponds.”

Your answer is correct. / Your answer is not correct. / Please give an answer to the next question!

2.3.8 Exercise 8

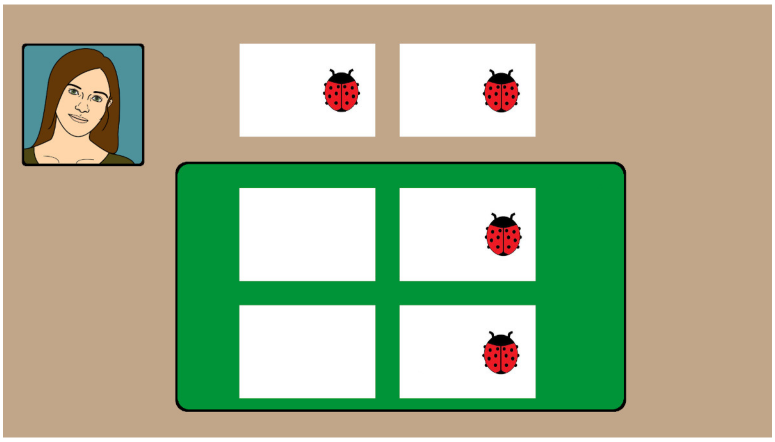

Lena: "Less than three cards on the table contain ladybugs."

Your answer is correct. / Your answer is not correct. / Please give an answer to the next question!

2.4 End of Exercises

This were the exercises. If you do not have any questions, the main experiment will start after pressing a button. Please note that you will not receive any feedback during the main experiment.

This part has 7 sections, in between you will have the opportunity to take short breaks.
